# Ubiquitous Transgenic Overexpression of C-C Chemokine Ligand 2: A Model to Assess the Combined Effect of High Energy Intake and Continuous Low-Grade Inflammation

**DOI:** 10.1155/2013/953841

**Published:** 2013-12-15

**Authors:** Esther Rodríguez-Gallego, Marta Riera-Borrull, Anna Hernández-Aguilera, Roger Mariné-Casadó, Anna Rull, Raúl Beltrán-Debón, Fedra Luciano-Mateo, Javier A. Menendez, Alejandro Vazquez-Martin, Juan J. Sirvent, Vicente Martín-Paredero, Angel L. Corbí, Elena Sierra-Filardi, Gerard Aragonès, Anabel García-Heredia, Jordi Camps, Carlos Alonso-Villaverde, Jorge Joven

**Affiliations:** ^1^Unitat de Recerca Biomèdica, Hospital Universitari Sant Joan, Institut d'Investigació Sanitària Pere Virgili, Universitat Rovira i Virgili, Carrer Sant Llorenç 21, 43201 Reus, Spain; ^2^Campus of International Excellence Southern Catalonia, Spain; ^3^Catalan Institute of Oncology and Girona Biomedical Research Institute, Avda de Francia s/n, 17007 Girona, Spain; ^4^Department of Pathology, Hospital Universitari Joan XXIII, C/ Dr. Mallafrè Guasch 4, 43005 Tarragona, Spain; ^5^Department of Vascular Surgery, Hospital Universitari Joan XXIII, C/ Dr. Mallafrè Guasch 4, 43005 Tarragona, Spain; ^6^Centro de Investigaciones Biológicas, Consejo Superior de Investigaciones Científicas, Ramiro de Maeztu 9, 28040 Madrid, Spain; ^7^Servei de Medicina Interna, Hospital Sant Pau i Santa Tecla, Rambla Vella 14, 43003 Tarragona, Spain

## Abstract

Excessive energy management leads to low-grade, chronic inflammation, which is a significant factor predicting noncommunicable diseases. In turn, inflammation, oxidation, and metabolism are associated with the course of these diseases; mitochondrial dysfunction seems to be at the crossroads of mutual relationships. The migration of immune cells during inflammation is governed by the interaction between chemokines and chemokine receptors. Chemokines, especially C-C-chemokine ligand 2 (CCL2), have a variety of additional functions that are involved in the maintenance of normal metabolism. It is our hypothesis that a ubiquitous and continuous secretion of CCL2 may represent an animal model of low-grade chronic inflammation that, in the presence of an energy surplus, could help to ascertain the afore-mentioned relationships and/or to search for specific therapeutic approaches. Here, we present preliminary data on a mouse model created by using targeted gene knock-in technology to integrate an additional copy of the CCl2 gene in the Gt(ROSA)26Sor locus of the mouse genome via homologous recombination in embryonic stem cells. Short-term dietary manipulations were assessed and the findings include metabolic disturbances, premature death, and the manipulation of macrophage plasticity and autophagy. These results raise a number of mechanistic questions for future study.

## 1. Introduction

Excessive energy intake is a part of the current human lifestyle that leads to a state of chronic systemic low-grade inflammation, which is thought to play a role in the development of atherosclerosis, cancer, and other noncommunicable diseases. At the same time, it is also plausible that the long-term consequences of prolonged inflammation exacerbate the deleterious effects of continuous nutrient surplus [1–3].

The immune system and metabolism are closely interconnected [4, 5]. During inflammation, the whole body is under metabolic stress, and energy excess management could compromise the relationships among metabolism, oxidation, and inflammation. We reasoned that searching for an adequate animal model [6] might allow us to better understand disease pathogenesis.

Chemokines are promising candidates for the design of such a model. Some of the functions of chemokines are associated with the migration of immune cells, and chemokines are important for the correct functioning of metabolism. In humans, C-C chemokine ligand 2 (CCL2; formerly referred as MCP-1 or monocyte chemoattractant protein-1) could be a marker of inflammation; it is overexpressed in noncommunicable diseases and is involved in a variety of metabolic functions [7]. Actually, CCL2 modifies lipid and glucose metabolism and contributes to insulin resistance and hepatic steatosis [8–11]. Of note, circulating chemokines cause and maintain metabolic disturbances that may be reversed by anti-inflammatory drugs, and the role of chemokines is likely a causal and predisposing factor [12, 13]. Rather than local overexpression [14–17], it is now recognised that CCL2 protein and mRNA are expressed in the vast majority of tissues, suggesting both a systemic production and the ability to respond *in situ* to inflammatory stimuli [18, 19].

Therefore, we hypothesised that challenging an animal model that systemically overexpresses CCL2 with diets rich in fat and cholesterol could help to assess the role of chronic inflammation in response to excessive energy intake. We then proceeded to integrate a copy of the *Ccl2* gene in the Gt(ROSA)26Sor (commonly referred to as ROSA26) locus of the mouse genome via homologous recombination in embryonic stem cells (ES) to generate targeted transgenic mice [20–22] that overexpress CCL2 in all tissues. Preliminary data are promising and suggest a number of mechanistic questions for future study.

## 2. Material and Methods

### 2.1. Animal Handling

All procedures and experimental protocols were examined and approved by the Ethics Review Committee for Animal Experimentation of the Universitat Rovira i Virgili. Basic protocols for tissue collection, diets, allocation concealment and metabolic assessment of the mice have been already described in detail [6, 18, 23]. Strains were backcrossed >10 generations to C57BL/6J mice and maintained homozygously. Littermates without mutations were used as controls (WT). We also provide data from knockouts (KO) of CCL2 (conveniently backcrossed), which were purchased from the Jackson Laboratory (Sacramento, CA). Dietary experiments began at 10 weeks of age, when all strains display similar phenotypes. To avoid possible effects of immature adipocyte modelling, most results were obtained in different groups after 6 or 14 weeks of treatment (16 and 24 weeksold, resp.). To explore dietary effects, mice from each group were fed either chow (Teklad rodent diet; Harlan, Barcelona, Spain) or a high-fat diet (FuttermittelfürMaüse; SSniff spezial diäten, Soest, Deutschland) and caged indefinitely under supervision. The breeding of all experimental populations was performed in our own facilities, and the progenies were maintained under close surveillance. The animals were not kept under germ-free conditions.

### 2.2. Targeted Transgenic (TG) Mice

**Figure 1:**
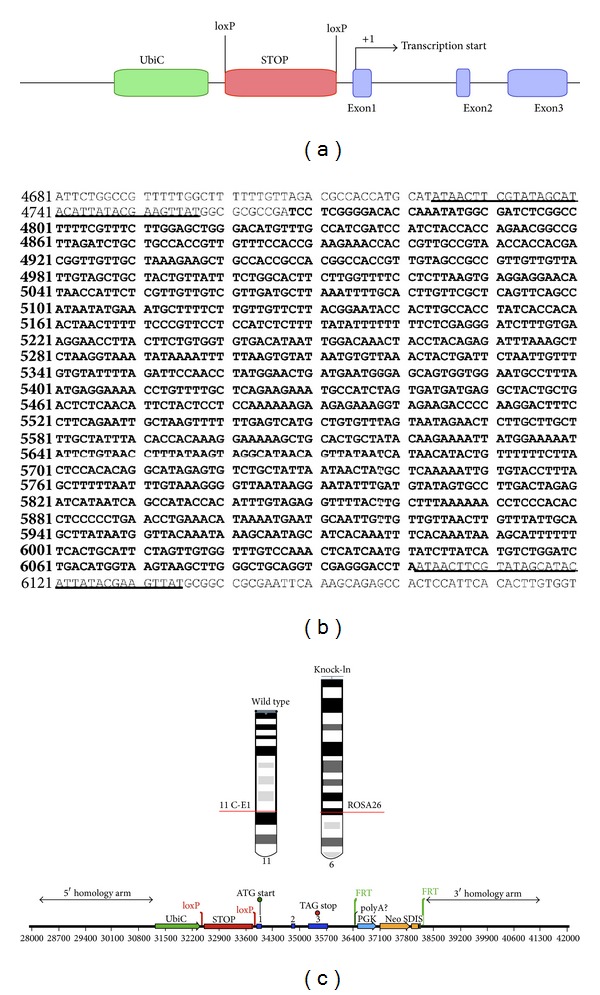
A STOP sequence flanked by loxP sites was inserted between the Ubiquitin promoter and the mouse *Ccl2* gene (a). The sequences of both the STOP cassette (bold) and the loxP sites (underlined) are shown later (b). The wild-type allele for *Ccl2* gene is located in the region 11 C-E1 of chromosome 11 and the transgenic vector (bottom) is inserted in the ROSA26 locus of chromosome 6 (c). The procedure is designed to avoid chromosomal instabilities.

The transgenic model was generated via a gene targeted inducible knock-in (KI), that is, a line with a duplicated gene, approach using standard methods and proprietary technology from Ozgene (Bentley, WA, Australia). The mRNA sequence corresponding to the mouse *Ccl2* gene (NM_011333 and ENSMUSG00000035385) is located on chromosome 11. The gene has 3 exons spread over approximately 3 Kb. The gene fragment was obtained from C57BL/6 genomic DNA (PCR primers AGCAAGATGATCCCAATGAGTAGGC and GAGGTGGTTGTGGAAAAGGTAGTGG) to be inserted by gene targeting into the ROSA26 locus. Upstream regulatory elements are important in the transcriptional regulation of *Ccl2* gene. Human ubiquitin promoter (Ubic) was chosen for the transgene to produce a high-level of expression. A loxP-flanked STOP cassette prevents the transcription of the gene following the UbiC promoter (See Figure 1 and Supplementary Materials S1 and S2 available online at http://dx.doi.org/10.1155/2013/953841). The STOP cassette can be removed using Crerecombinase. PGK-Neo-SD-IS, a selection cassette, is inserted downstream of the *Ccl2* gene to enrich homologous recombination events. The ROSA26 locus is conserved between mice and humans. The location is autosomal (chromosome 6) and is actively transcribed in most tissues (Figure 1). Moreover, epigenetic inactivation is unlikely [21, 24–26].

The combination of gene targeting and ES cell technology exploiting homologous recombination provides advantages over other techniques [27–31] (Supplementary Material S3). Mice are available upon request.

### 2.3. Immunopathology Studies and Assessment of Liver Steatosis

Portions of organs and tissues were either frozen in nitrogen or fixed in 4% phosphate-buffered formalin for 24 h at room temperature, washed twice with water, stored in 70% ethanol at 4°C, and embedded in paraffin for histological analyses. Primary and secondary antibodies were obtained from Santa Cruz Biotechnology (Heidelberg, Germany) and Serotec (Oxford, UK) [18, 32]. Detection was performed with the ABC peroxidase system (Vector, Burlingame, CA) using DAB (Dako, Glostrup, Denmark) as the substrate. To assess specificity, primary antibodies were omitted in the controls. Liver steatosis was assessed as previously described [6].

### 2.4. Laboratory Measurements

We measured murine CCL2 in plasma, serum, and tissues by ELISA (Peprotech, London, UK), according to the instructions of the manufacturer. Recombinant human CCL2 antigen was used as the calibrator for assay standardisation, and we found weak cross-reactivity with other chemokines, especially CCL7. The intraassay coefficients of variation were <3.2%, and the interassay of variation was <9.1%. Other biochemical measurements were performed in automated analysers using commercially available reagents as described [6, 33]. Selected tissues were homogenised using the Precellys 24 system (Izasa, Barcelona, Spain) with prefilled bead tubes in the buffer of choice. Fractions of the homogenised liver were immunoblotted as described [34], using antibodies and reagents from Santa Cruz Biotechnology (Heidelberg, Germany).

### 2.5. Transmission Electron Microscopy

Small pieces of the liver were immediately fixed in a 2% glutaraldehyde solution in 0.1 M cacodylate buffer, pH 7.4. Samples were then postfixed in 1% osmium tetroxide (OsO_4_) for 2 h and dehydrated in sequential steps of acetone prior to impregnation in increasing concentrations of the resin in acetone over a 24 h period. Semithin sections (500 nm) were stained with 1% toluidine blue. Ultrathin sections (70 nm) were subsequently cut using a diamond knife, double-stained with uranyl acetate and lead citrate, and examined using a transmission electron microscope (Hitachi, Tokyo, Japan).

### 2.6. Characterisation of Mouse Bone Marrow-Derived Macrophages

The methods were performed as previously described [35]. Bone marrow cells were isolated by removing leg bones from WT and TG mice (aged 10 weeks) and were cultured for 24 hours. Floating cells were removed, and the remaining attached cells were analysed. Cells were further cultured in DMEM supplemented with 10% inactivated foetal calf serum, 50 *μ*M beta-mercaptoethanol, and 1000 U/mL murine granulocyte-macrophage colony-stimulating factor (GM-CSF) or 25 ng/mL human macrophage colony-stimulating factor (M-CSF) (ImmunoTools, Friesoythe, Germany) to provide polarised activation of cells into M1 and M2 as a simplified descriptor of their functional plasticity. To assess the effect of activation, macrophages were treated with 100 ng/mL *E. coli* 055:B5 lipopolysaccharide (LPS) for 24 hours and were compared with the respective untreated controls. After this treatment, supernatants from M1 (GM-CSF) and M2 (M-CSF) macrophages were tested for the presence of CCL2, tumour necrosis factor-*α* (TNF*α*), and interleukin 10 (IL-10) using ELISA (BioLegend, Inc., Madrid, Spain). Total RNA was extracted using the RNeasy kit (Qiagen, Barcelona, Spain) and was retrotranscribed using the Reverse Transcription System kit (Applied Biosystems; Invitrogen, Barcelona, Spain). Oligonucleotides for selected genes were designed according to the Roche software for quantitative real-time PCR (Universal Probe Roche library), which was performed using a LightCycler 480 (Roche Diagnostics, Barcelona, Spain). The assays were performed in triplicate, and the results normalised according to the expression level of TATA-binding protein mRNA. C-C chemokine receptor type 2 (CCR2 or CD192), TNF*α*, inhibin beta A (INHBA), inducible nitric oxide synthase (iNOS), C-C chemokine receptor type 7 (CCR7), and Egl nine homolog 3 (EGLN3) were chosen as M1 markers. Arginase (ARG), EMR1/F4/80, insulin growth factor-1 (IGF1), IL-10, the mannose receptor CD206, and growth arrest-specific 6 (GAS6) were chosen as M2 markers.

### 2.7. Statistical Analyses

The normality of the distributions was assessed using the Kolmogorov-Smirnov method. Variables were compared using Mann-Whitney tests or Kruskal-Wallis one-way analysis adjusted for multiple testing. Unless otherwise indicated, the values in the figures represent the mean and SEM obtained in groups of 8 mice. The *χ* ^2^ test was used to compare categorical variables. For all measurements, we used either SPSS (SPSS Inc., Chicago, IL) or GraphPad Prism software (http://www.graphpad.com/scientific-software/prism/).

## 3. Results

### 3.1. Targeted Transgenic Mice Do Not Display Physical Abnormalities

**Figure 2:**
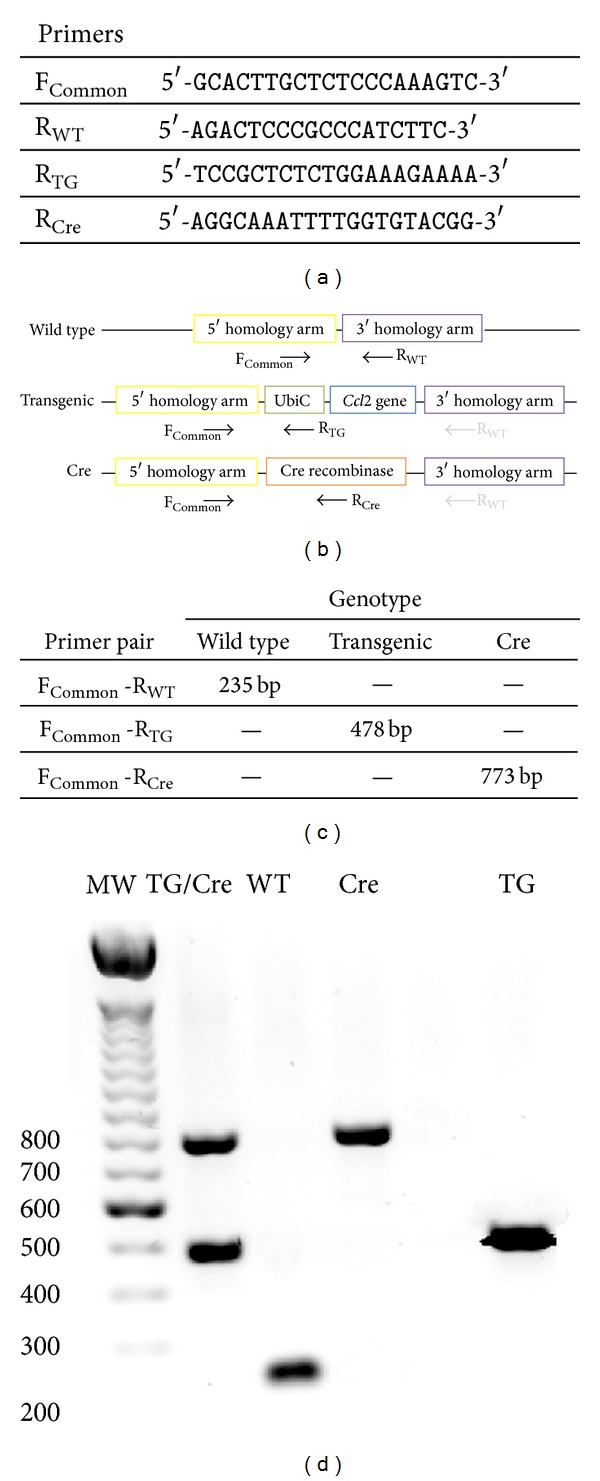
Simplified strategy for genotyping that includes the sequence of each primer (a), the reaction proposed for each primer (b), and the expected PCR products for each strain (c). The method is designed for the concomitant use of all primers and a representative gel is shown in (d).

The resulting mice for the targeted mutation are viable, fertile, and normal in size and weight. The animals do not display apparent behavioural or reproductive defects. The transgene insertion of a single copy occurs at a defined site, which allows for easy genotyping (Figure 2) and eliminates possible instabilities, independent segregation during breeding, and unpredictable positions in the chromosomes.

An additional advantage of this strategy is the Cre/lox recombination system that facilitates tissue-specific overexpression. The Ubic is conditioned by an Lox-Stop-Lox (LSL) element that is activated by Cre-mediated excision using the appropriate, tissue-specific Cre strain.

### 3.2. Transgenic Mice Overexpress CCL2 in Selected Tissues, and Circulating Protein Is Increased with respect to Controls

**Figure 3:**
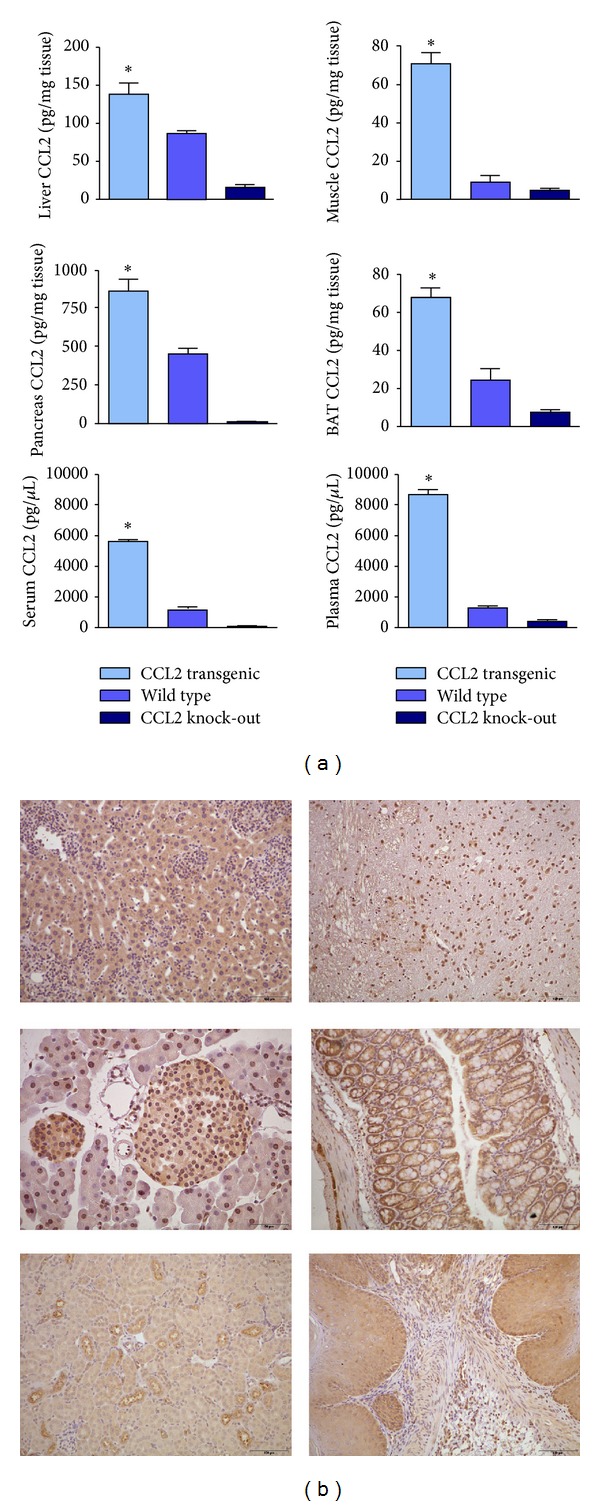
Overexpression of CCL2 with respect to wild type and knockout was observed in all selected tissues (extracts) in the transgenic mice as measured by ELISA. Differences were also observed in plasma and serum and there was cross-reactivity with similar chemokines that could explain the detection of CCL2 in KO mice (a). CCL2 was also detected by immunochemistry in different types of cells (b). *P < 0.005; Micrographs in the left column are representative for liver, pancreas, and kidney. Those in the right column were for brain, intestine, and stomach.

Consistently, transgenic mice displayed more CCL2 protein in all tissues examined with respect to WT animals. The differences increased with age, and there were minor relative differences among tissues (Figure 3). We confirm that CCL2 was immunologically detected in all selected tissues of the transgenic mice. The CCL2 mRNA expression in the transgenic mice was also higher in different types of cells with respect to WT mice. The amount of CCL2 expression was higher after the designed period of exposure to a diet with a high fat content. Of note, the serum and plasma CCL2 were also higher in transgenic mice than in WT mice, which is most likely caused by CCL2 secretion by multiple tissues. In accordance with previous observations, the plasma concentrations differed from the serum concentration. The differences are likely caused by coagulation and handling, but the differences were not statistically significant in transgenic mice. Notably, CCL2 was also detected in KO mice, but with less intensity. This is most likely due to quantitatively minor cross reactivity, as described in the methods.

### 3.3. Dietary Factors Influence Body Weight and Adipocyte Size

**Figure 4:**
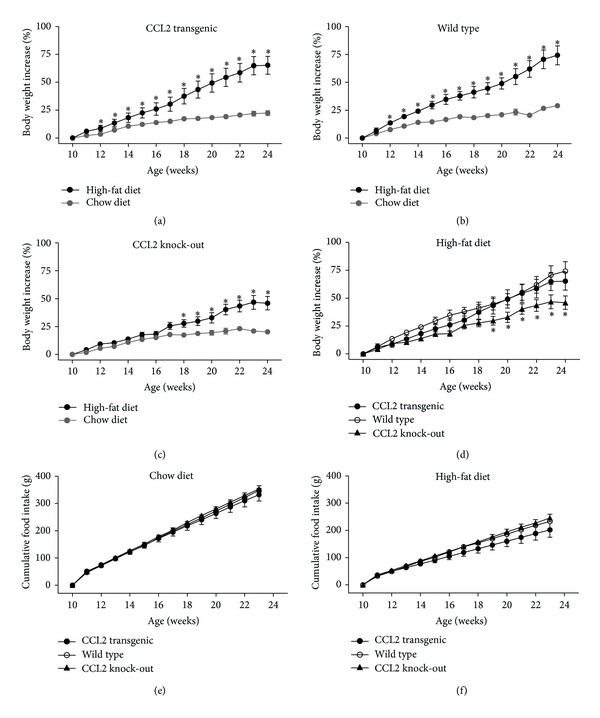
The effect of high-fat diet in body weight increase was evident in transgenic and wild-type mice ((a), (b)), but the different increase was immediate after dietary manipulation in transgenic. This effect was negligible in knockout mice (c). The combination of these effects with high-fat diet (d) shows similar results to facilitate comparison. These findings are not due to differences in the cumulative food intake ((e), (f)) indicating that CCL2 probably has no effect on appetite. *P < 0.05.

When mice were fed a regular chow diet, we did not observe significant differences in body weight increase among groups. The cumulative food intake was identical for the three strains examined. In contrast, when fed a high fat diet, both transgenic animals and WT animals developed obesity. Of note, the C57BL/6J male mouse is a commonly used model of diet-induced obesity [36]. The effect of CCL2 overexpression was apparent immediately after the ingestion of the high calorie diet, and the weight increased more rapidly than in WT mice. The absence of CCL2, however, protected the KO mice from excessive weight gain. The lack of significant differences in the food intake excluded any effect of CCL2 on appetite (Figure 4).

**Figure 5:**
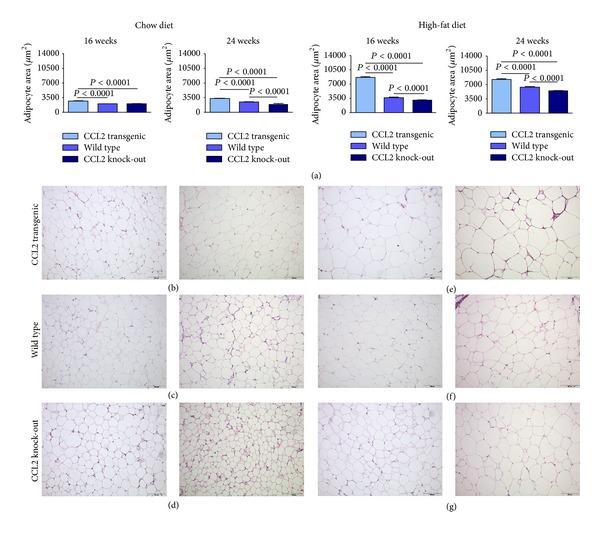
The size of the adipocytes was significantly higher in transgenic mice than in wild-type and knockout mice and the effect was observed with both dietary interventions regardless of the duration of the dietary treatment (6 or 14 weeks) (a) but it was more intense when mice were fed a high-fat diet. For clarity, values are indicated only for adipocytes in epididymal white adipose tissue. Representative micrographs are shown for transgenic, wild-type, and knockout animals ((b), (c) and (d), resp.) when fed a chow diet and for the corresponding animals fed a high-fat diet ((e), (f), (g)) at 16 and 24 weeks' old.

**Figure 6:**
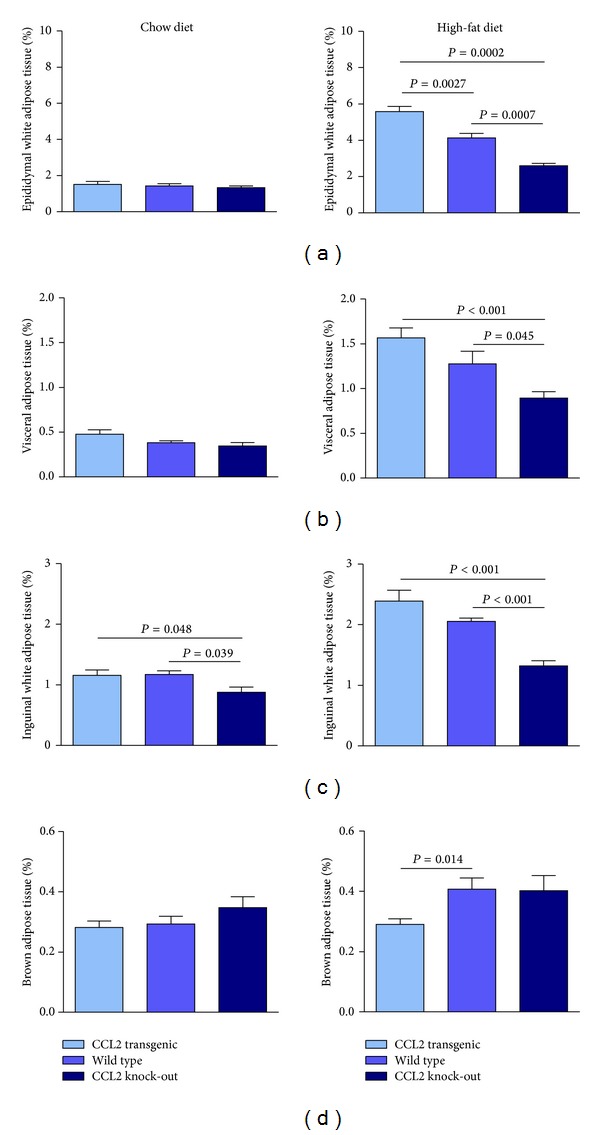
The effect of CCL2 expression in the weight of adipose tissue ((a)–(d)) of animals fed either chow (left column) or high-fat diet (right column). Of note, differences among strains were more evident during energy surplus and no change was observed in brown adipose tissue.

Overexpression of CCL2 also increased the size of the adipocytes. Data are presented for epididymal adipose tissue (Figure 5), but the effect was similar in other adipose tissues. The adipocyte size was significantly higher in CCL2 transgenic animals compared with WT and KO animals fed with both diets, but the difference was higher when mice were fed a diet with a high caloric content. When different types of adipose tissue were weighed, we found that the mice fed a chow diet showed no significant differences between the strains, with the possible exception of inguinal tissue. Conversely, the addition of fat to the diet resulted in a significant increase in the weight of white adipose tissue from other depots in mice with CCL2 overexpression. Notably, there was no effect on the weight of brown adipose tissue (Figure 6 and Supplementary Material S4). However, these differences among groups in adipose tissues weight disappeared when mice were fed with a high fat diet for 14 weeks. These results are probably indicating an already reported effect of adipose tissue remodelling on the consequences of high-fat dietary intake [37] (Supplementary Material S5).

### 3.4. Diet-Induced Disturbances in Glucose and Lipid Metabolism

Glucose tolerance tests (a proxy for insulin resistance) were unaffected in strains fed the chow diet during the experimental period of 6 weeks. However, WT littermates, KO, and transgenic mice displayed abnormal values when fed a high-fat diet, confirming the effect of diet in the pathogenesis of insulin resistance and suggesting that this short-term intervention is not adequate to investigate a possible role, if any, of CCL2 in the generation of glucose and lipid disturbances. Moreover, there were no differences among the strains in the plasma glucose levels after 6 hours of fasting, and after 3 hours in the fasting state, we found that the plasma glucose baseline concentrations were significantly higher in CCL2 overexpressing mice with respect to CCL2 deficient animals. This effect was more evident in the transgenic mice (Supplementary Material S6) but differences in plasma glucose disappeared after 14 weeks of dietary treatment suggesting immature adipose tissue remodelling [38].

When these tests were performed in animals fed a high-fat diet for a longer experimental period of 14 weeks in which adipose tissue is already well modelled, the lack of differences in insulin tolerance was maintained, probably indicating that the effect of CCL2 overexpression in the pathogenesis of insulin resistance is negligible.

However, results in the absence of CCL2 indicate that this chemokine may modify glucose metabolism and therefore we cannot discard the effects under a more intense metabolic stress [9]. Variations in plasma cholesterol and triglycerides concentrations were minimal among the strains at 16 weeks old. A high-fat diet significantly increased the amount of circulating cholesterol, an effect that was higher in CCL2 overexpressing mice. Conversely, there were unexpected, and most likely not representative, changes in the plasma triglycerides concentration of these mice as a consequence of dietary manipulations (data not shown).

### 3.5. The Influence of CCL2 and Dietary Manipulations in the Liver

**Figure 7:**
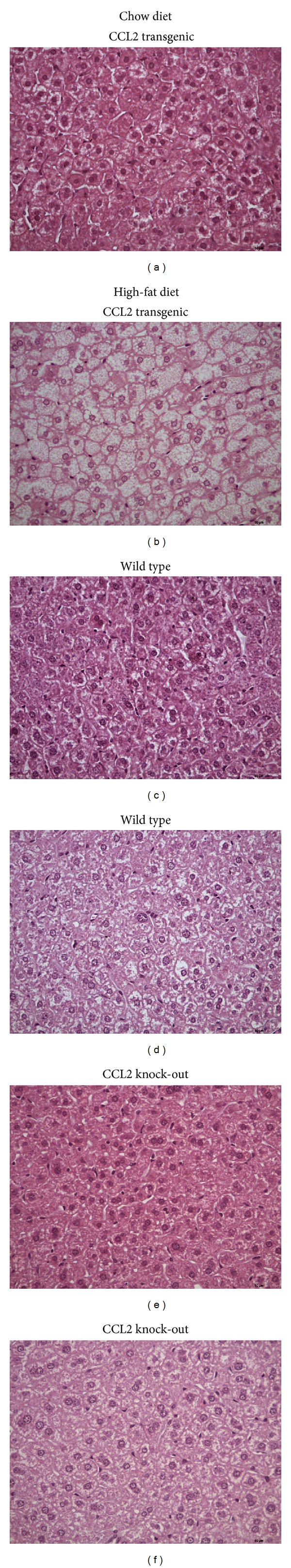
We found no significant differences among strains in the appearance of liver tissue when mice were fed a chow diet (left column (a), (c), (e); transgenic, wild-type, and knockout mice resp.). Representative micrographs show in the right column that a high fat diet produces steatosis in transgenic mice (b), dispersed lipid droplets in the liver of wild type mice (d), and no change in knockout mice (f).

When fed the chow diet, mice did not display significant differences among strains in the appearance of their liver tissue. The steatosis scores did not detect significant differences among strains, although some minor variations were detected (Figure 7) that did not correlate with the hepatic lipid content (data not shown). When mice were fed a high-fat diet, we found a certain amount of lipid accumulation in WT mice, but this lipid accumulation was significantly more evident in transgenic mice. Conversely, there was no accumulation of lipids in KO mice (Figure 7). Therefore, the effect of CCL2 under these conditions is directly related to the amount of tissue CCL2 disposal; the absence of CCL2 prevents liver steatosis, and overexpression of CCL2 predisposes the liver to steatosis. We also found that the expression of fatty acid synthase in the liver increased significantly in all strains when fed a high-fat diet, but there were no significant differences in the comparisons between transgenic and KO mice. We also explored the activating phosphorylation of AMP-activated protein kinase (AMPK), and values did not change as a result of high-fat diet in transgenic mice and were significantly higher in KO mice compared with transgenic mice (Supplementary Material S7).

**Figure 8:**
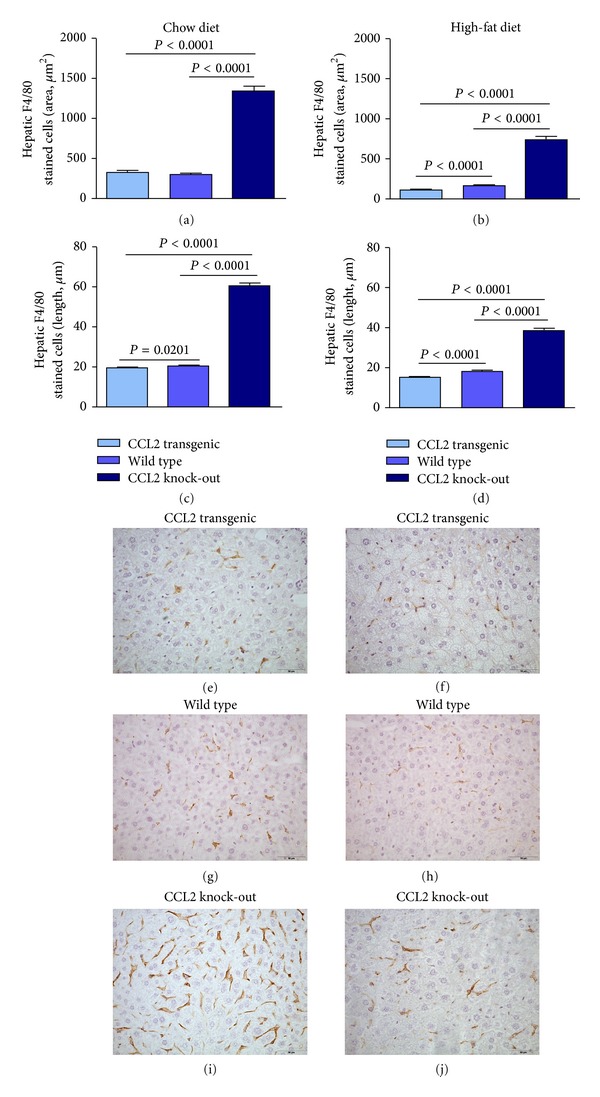
Dietary fat (right column) and CCL2 expression modify the size, number, and morphology of liver macrophages with respect to those fed a chow diet (left column) as assessed with F4/80 staining. Values for stained area and length of macrophages ((a)–(d)) are illustrated with representative microphotographs from transgenic ((e), (f)), WT ((g), (h)) and KO mice ((i), (j)).

**Figure 9:**
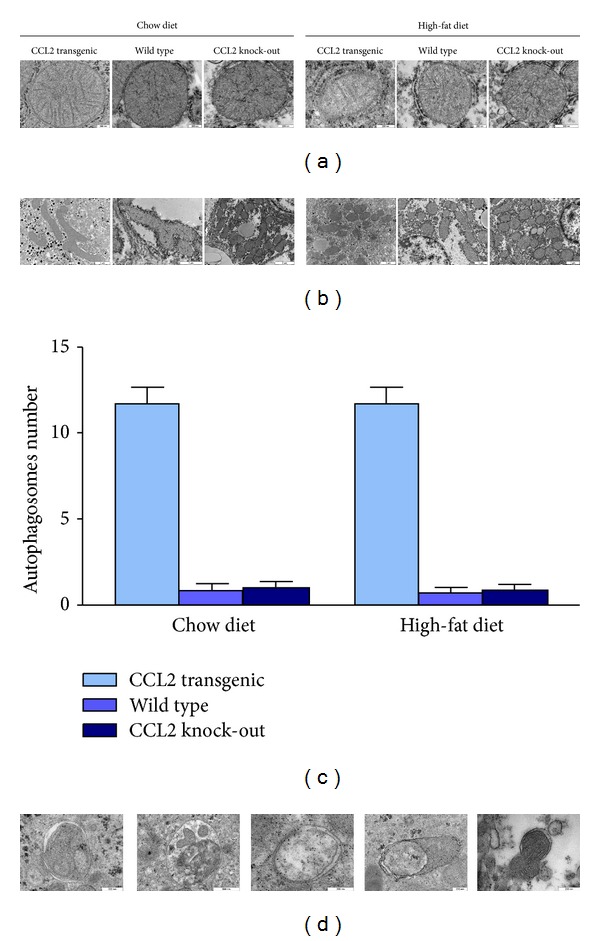
The appearance of mitochondria was affected by the dietary manipulation and the expression of CCL2 as shown in representative microphotographs (a) and these changes were accompanied by a significant effect in fusion-fission balance (b). The number of autophagosomes per cell was counted and was significantly higher in transgenic mice. Further, these were rare in both WT and KO and independent of diet (c). The heterogeneous nature of autophagic elements is illustrated in (d) (photographs obtained in transgenic mice).

When the livers were examined for the presence of F4/80 antigen, a widely accepted marker of macrophages, we found that both dietary fat and overexpression of CCL2 modify the size, number and morphology of liver macrophages (Figure 8 and Supplementary Material S8). Of note, F4/80 stained cells were more frequent in KO mice, a finding that merits further study because these results could represent a change in function and could be responsible for the differential effects of CCL2 in liver steatosis. We then explored the influence of both CCL2 and diet in mitochondrial biogenesis. Based on the appearance of the matrix, the mitochondria are healthier in mice fed a chow diet than in those fed a high-fat diet. The matrix was also consistently less electron-dense in transgenic mice. We also found altered fusion dynamics. In transgenic mice fed a chow diet, the process was unbalanced towards mitochondrial fusion, but the dietary manipulation significantly elicited a shift towards fission. The changes were similar in WT mice, but the effect of diet was quantitatively less evident than in transgenic mice. In KO mice, however, there were more mitochondria per cell, and fusion and fission were correctly balanced and apparently not altered by differences in diet. These findings strongly support further mechanistic studies, which may link the expression of CCL2 with mitochondrial biogenesis, inflammation, and energy management. According to our results, these putative mechanisms are related to the autophagic response, which was clearly enhanced in transgenic mice. Conversely, most liver cells in WT and KO displayed no evidence of autophagy (Figure 9).

### 3.6. Transgenic Mice That Overexpress CCL2 Die Prematurely When Fed High-Fat Diet

The transgenic mice fed a high-fat diet died prematurely between 10 and 14 months. The mice progressively decreased activity, reduced food intake and the appearance of frailty became evident. There was also a casualty in the transgenic mice fed chow diet, but it was sudden, unexpected, and without a prior decrease in weight or activity. Among the casualties, one was also observed in the WT group fed a high-fat diet (Supplementary Material S9). A full autopsy was performed, and the cause of death was uncertain. There was neither cancer nor arteriosclerosis in these animals, but there were some cutaneous, superficial, and localised lesions in the skin accompanied with local loss of hair. There was also no evidence of sepsis. The only remarkable findings were limited to the spleen and the liver. The size and weight of the spleen was consistently higher in the transgenic mice fed high-fat diet. The presence of splenomegaly in these transgenic mice was consistent with the presence of giant cells that were identified as megakaryocytes (Factor VIII positive staining) and other proliferative signs. The weight of the liver was also higher in the transgenic mice, which is most likely due to the higher presence of steatosis. In the liver, there were signs of regenerative cells and increased apoptosis. Ongoing studies with higher sample sizes and the inclusion of females have been designed to further ascertain this point.

### 3.7. Bone Marrow Macrophages of Transgenic Mice: Expression of Selected Cytokines and mRNA

**Figure 10:**
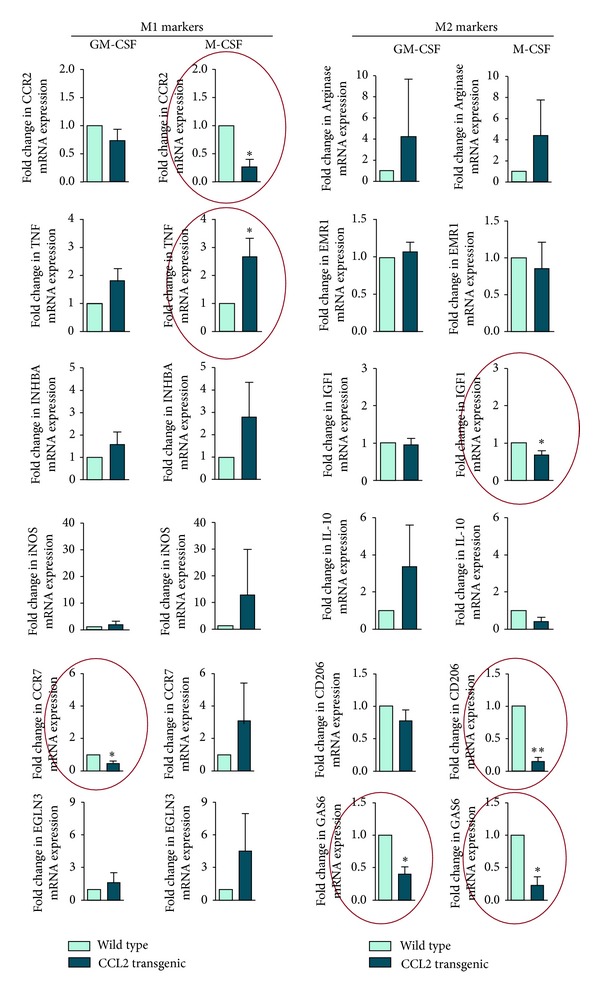
Relative mRNA expression in transgenic mice with respect to WT mice of selected markers for M1 and M2 macrophages in cells treated *in vitro* with either GC-MSF or M-CSF. Acronyms used were C-C chemokine receptor type 2 (CCR2), TNF*α*, Inhibin, beta A (INHBA), inducible nitric oxide synthase (iNOS), C-C chemokine receptor type 7 (CCR7), Egl nine homolog 3 (EGLN3), Arginase (ARG), EGF module-containing mucin-like hormone receptor EMR1 (F4/80), insulin growth factor-1 (IGF1), IL-10, the mannose receptor CD206, and Growth arrest-specific 6 (GAS6).

**Figure 11:**
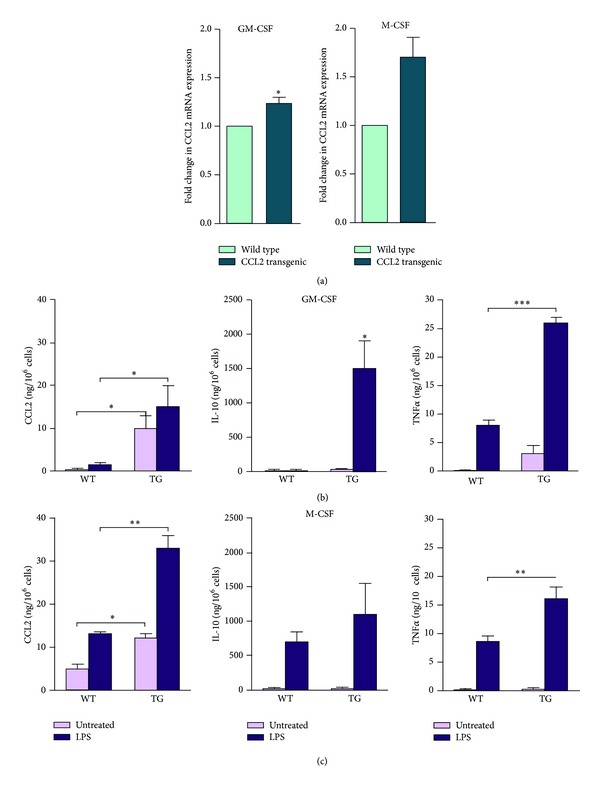
The relative CCL2 mRNA expression (a) and the secretion in supernatants of selected cytokines in bone marrow-derived macrophages of transgenic and WT cells treated *in vitro* with either GC-MSF (b) or M-CSF (c).

The CCL2 mRNA expression in the bone marrow macrophages was higher in transgenic than in WT mice, irrespective of stimulation with either GM-CSF (M1, pro-inflammatory) or M-CSF (M2, phagocytic). The mRNA expression of the selected M2 markers was similar, with either low or undetectable expression in the GM-CSF macrophages without differences between transgenic and WT mice. The expression of the selected M1 markers was practically identical in the GM-CSF macrophages from TG and WT mice, with the notable exception of CCR7. Surprisingly, the expression of this chemokine receptor was significantly lower in TG mice, indicating lower pro-inflammatory activity. The expression of the M1 markers in M-CSF macrophages showed a unique and significant decrease in CCR2 mRNA expression; however, some M2 markers, including CD 206, GAS6, and IGF1, were also underexpressed. IL-10 expression also decreased, but the differences were not statistically significant. The results suggest that CCL2 overexpression may alter macrophage polarisation. Consequently, the secretion of selected cytokines was examined in macrophages that were treated with LPS and were compared with the relevant controls. The CCL2 secretion was higher in TG mice with both treatments compared with the WT mice and was 2–4 fold higher (2–4-fold change) in M-CSF macrophages. The IL-10 secretion was clearly detectable only in LPS-treated animals. The concentration in the supernatant was higher in TG than in WT mice, and the differences were statistically significant in GM-CSF macrophages. Finally, TNF*α* secretion was ostensibly higher in LPS-treated animals and significantly higher in TG mice with respect to the relevant controls (Figures 10 and 11).

## 4. Discussion

The transgenic mice developed in this study systemically overexpress CCL2. These animals were created to assess the combined effect of the recruitment of circulating monocytes in all tissues and the response to the stimuli of high dietary fat and energy ingestion. The hypothesis was that the continuous overexpression of this chemokine could promote or worsen common pathological conditions, and as animal model could be useful for assessing the pathogenic mechanisms and therapeutic approaches [39].

Fertility, growth, and physical appearance were identical to the controls. CCL2 overexpression did not result in abnormalities in the mice that were fed a regular chow diet. However, adding fat to the diet during a short period of time caused differences in body weight, adipocyte size, disturbances in glucose and lipid metabolism, premature death, and liver alterations that included a higher predisposition to fatty liver disease and significant changes in mitochondrial biogenesis and autophagy. Additionally, we explored bone marrow macrophages under different *in vitro* conditions, and we found that CCL2 overexpression affects functional plasticity.

In previous studies, CCL2 has been considered a chemokine secreted by adipose tissue (adipokine), but systemic CCL2 overexpression regulates white adipose tissue (WAT) mass and size without apparent effects in brown adipose tissue (BAT). WAT serves primarily as lipid storage, and BAT is used for heat generation. The balance between the two adipose tissues affects the whole-body energy homeostasis, and the development and severity of obesity [40]. A higher production of CCL2 is not only a consequence of obesity but is most likely an exacerbating factor of diet-induced alterations. The roles of CCL2 in the aetiology of obesity and diabetes, the regulatory mechanisms, and the effect of therapies that inhibit CCL2 production have been recently reviewed [4, 41]. We have also found differences between fat cells in different adipose tissue depots and the heterogeneity of adipocytes within the same depots. Further examination of this issue is necessary because a different pattern of gene expression could explain the differential development of various types of adipose tissue [42, 43]. Moreover, this is closely associated with the pattern of fat distribution, the extent of obesity, and consequently the impact of different fat depots on the severity of metabolic complications [44, 45].

The size and number of hepatic macrophages significantly differs between transgenic and KO mice when detected with antibodies directed against F4/80. Curiously, this is an extracellular antigen of unknown function that belongs to a subgroup of the G-protein-coupled receptors [46]. The changes in macrophages morphology could represent concomitant changes in function and whether the macrophages are resident or recruited. This is further substantiated by the fact that these transgenic mice were prone to develop fatty liver disease and the KO mice were protected. The role of increased CCL2 is not yet understood, but the recruitment of macrophages seems to be important in different animal models. In KO mice there is an increased expression of peroxisome proliferator-activated receptors accompanied by the induction of fatty acid metabolism-related genes and the inhibition of pro-inflammatory cytokine production [47–49]. We confirmed that the effect of fat in the pathogenesis of fatty liver disease [49, 50] is influenced by the amount of available CCL2 and that the linkage between chemokines and hepatic lipid metabolism is plausible.

The characterisation of bone marrow-derived cells in the transgenic mice indicates that CCL2 overexpression affects the transition in the secretory function of macrophages (or the M1-M2 paradigm as a simplified descriptor of the functional plasticity). This is illustrated by differences in GM-CSF and M-CSF, which are cytokines that differentiate macrophages *in vitro* with distinct morphology and inflammatory function [51, 52]. The modulation of the phenotypic and functional differences in macrophage polarisation by CCL2 overexpression denotes a shift towards lower pro-inflammatory activity [53]. CCL2 decreased the expression of CCR7 in M1 and decreased the expression of CCR2, IGF1, CD206, IL-10, and GAS6 in M2. In cells under LPS treatment, however, CCL2 overexpression increased the secretion of IL-10 and TNF*α* with respect to WT controls. These changes could represent a quantitatively determinant factor in the development of macrophage-induced metabolic alterations. It should be highlighted that a high percentage of total body resident macrophages are present in the liver and that adipose tissue is a major site for the accumulation of recruited macrophages [54, 55].

Notably, CCL2 is involved, directly and/or through the induced metabolic alterations, in mitochondrial biogenesis and autophagy. We add CCL2 to the growing list of nonessential regulators of mechanisms that divide and fuse mitochondria [56]. The balance between rejuvenation and elimination of damaged mitochondria via autophagy is affected by both the presence of CCL2 overexpression and the increased availability of energy. The antagonistic and balanced activities of the fusion and fission machineries are constantly providing responses to inflammation to tightly regulate homeostasis of the organism [57, 58]. This is expected because mitochondrial diseases are associated with metabolic alterations. Apparently, there is a shift towards fusion in CCL2 overexpression to maximise ATP synthesis. Contrarily, morphological findings in CCL2 deficient mice, which are independent of high-fat diet, suggest a perfect balance [59, 60]. A certain unbalance is expected in inflammatory conditions and other energy-dependent disturbances via mitochondrial dysfunction [61, 62]. This is important because mitochondria and the access to energy (calorie restriction or increased dietary fat) play a pivotal role mediated by the mechanistic target of Rapamycin (MTOR) in deciding whether liver cells live or die [63].

In transgenic mice, autophagy was increased with respect to WT and KO mice, which is particularly important because autophagy affects immune responses as a result of degradative, biogenetic, and secretory activities that respond to various inputs via MTOR [64, 65]. Autophagy might control the infection of certain pathogens but also prevents excessive inflammatory reactions in the host [66]. As shown in autophagy-deficient macrophages, autophagy removes a number of proinflammatory stimuli [67–69]. Therefore, increased liver autophagy during CCL2 overexpression could be interpreted as an effort from the host to avoid the deleterious action of continuous inflammation.

Links between autophagy and inflammation have also been found in immune functions affecting several diseases, opening a new dimension in the understanding of the multifactorial basis of noncommunicable diseases. For example, increasing macrophage autophagy protects patients with advanced atherosclerosis [70]. It has also been reported that CCL2 controls the extent of autophagy in human prostate cancer [71], and autophagy is pivotal for the survival and differentiation of monocytes [72].

Finally, CCL2 overexpression resulted in premature death when combined with a high-energy intake. These findings require more extensive examination, and the cause of death remains obscure. Mice progressively lost interest in the environment, reduced activity, and their intake of food decreased. No chronic disease was evident, and there were no signs of sepsis or major infection. It is tempting to consider the possibility of premature aging, and future investigations will include the characterisation of a senescence-associated secretory phenotype, particularly in pro-inflammatory cytokine enrichment [73] and the pro-inflammatory phenotype that accompanies aging [74, 75].

## 5. Conclusions, Perspectives, and Limitations

This animal model raises a number of questions about the prevalent diseases responsible for limiting the quality of modern life. Additionally, this model provides a link between inflammation and metabolism and suggests targets for the management of diseases in which there is a clear CCL2 overexpression. Specifically, this model can help to uncover the role of CCL2 in mitochondrial dysfunction, autophagy, and functionality of macrophages and aging in combination with excessive energy intake. Information gained could be useful for designing new mechanism-based therapeutic strategies.

None of the described effects appear in mice that are fed a regular diet, and this fact highlights the importance of calorie restriction for health. Therefore, the nutrient-sensing MTOR pathway seems to be crucial for the management of noncommunicable diseases. Consequently, drugs modulating MTOR are obvious candidates for assessment. For example, experiments on cancer, aging, and viral infections strongly suggest that this is the case for metformin [76–78]. This antidiabetic drug activates AMPK and inhibits MTOR with potent antiinflammatory actions. The usefulness of rapamycin, an MTOR inhibitor, and similar drugs in cancer prevention has been assayed [79]. Aspirin decreases inflammation, inhibits the MTOR pathway, decreases cancer incidence, and may reduce the burden of atherosclerosis [13, 80]. Lastly, although studies are scarce, angiotensin-II-blockers and beta-blockers, widely used in hypertensive patients, can also prevent the activation of the MTOR pathway and the incidence of chronic diseases [81].

The potential indications for these drugs are mostly related to chronic diseases in which inflammation plays a crucial role. This animal model could be used to further select candidates and suggests a number of mechanistic questions for future study. Particularly, we consider this model as a valuable contribution to our evolving comprehension of the interphase between autophagy and inflammation. However, we acknowledge that care must be taken in analysing the results of studies performed in animal models and that further research effort is necessary to fully characterize our observations. To name a few, possible effects of sex should be studied and metabolic alterations should be confirmed with the use of metabolic cages and more specific methods to detect significant differences. Particularly, CCL2 may have a higher influence if there is a relative contribution from different type of cells, particularly from immune cells [72].

## Supplementary Material

Supplementary Material S1: comparison between human and murine CCL2 regions, corresponding to the manuscript.
Supplementary Material S2: details of the sequence of other main elements used in the construction of the vector, corresponding to the manuscript.
Supplementary Material S3: details on the construct design, corresponding to the manuscript.
Supplementary Material S4: Weight of selected tissues and organs, corresponding to the manuscript.
Supplementary Material S5: Weight of adipose tissues in the three strains after 14 weeks of a chow diet or high fat diet treatment.
Supplementary Material S6: disturbances in glucose metabolism, corresponding to the manuscript.
Supplementary Material S7: expression of fatty acid synthase and AMPK in the liver, corresponding to the manuscript.
Supplementary Material S8: dietary fat and overexpression of CCL2 modify the size, number and morphology of liver macrophages also after 14 weeks of dietary treatment corresponding to the manuscript.
Supplementary Material S9: Transgenic mice that overexpress CCL2 die prematurely when fed high-fat diet, corresponding to the manuscript.
