# Supplementary material for: Ubiquitous Transgenic Overexpression of C-C Chemokine Ligand 2: A Model to Assess the Combined Effect of High Energy Intake and Continuous Low-Grade Inflammation
Source: Mediators Inflamm. 2013 Dec 15;2013:953841. doi: 10.1155/2013/953841 (PMC3876923; doi:10.1155/2013/953841)
Supplement: Supplementary file 1 — Supplementary Material S1: comparison between human and murine CCL2 regions, corresponding to the manuscript. Supplementary Material S2: details of the sequence of other main elements used in the construction of the vector, corresponding to the manuscript. Supplementary Material S3: details on the construct design, corresponding to the manuscript. Supplementary Material S4: Weight of selected tissues and organs, corresponding to the manuscript. Supplementary Material S5: Weight of adipose tissues in the three strains after 14 weeks of a chow diet or high fat diet treatment. Supplementary Material S6: disturbances in glucose metabolism, corresponding to the manuscript. Supplementary Material S7: expression of fatty acid synthase and AMPK in the liver, corresponding to the manuscript. Supplementary Material S8: dietary fat and overexpression of CCL2 modify the size, number and morphology of liver macrophages also after 14 weeks of dietary treatment corresponding to the manuscript. Supplementary Material S9: Transgenic mice that overexpress CCL2 die prematurely when fed high-fat diet, corresponding to the manuscript. [file 953841.f1.pdf]

Supplementary material S1, comparison between human and murine CCL2 regions, corresponding to the manuscript:

Ubiquitous transgenic overexpression of C-C chemokine ligand 2: a model to assess the combined effect of high energy intake and continuous low-grade inflammation?

Esther Rodríguez-Gallego, Marta Riera-Borrull, Anna Hernández-Aguilera, Roger Mariné-Casadó, Anna Rull, Raúl Beltrán-Debón, Fedra Luciano-Mateo, Javier A. Menéndez[1], Alejandro Vazquez-Martin[1], Juan J. Sirvent[2], Vicente Martín-Paredero[3], Angel L. Corbí[4], Elena Sierra-Filardi[4], Gerard Aragonès, Anabel García-Heredia, Jordi Camps, Carlos Alonso-Villaverde[5], Jorge Joven*

A, displays a schematic view of human *CCL2* gene region that includes its regulatory region and the most prominent elements identified. The *Ccl2* gene transgenic vector includes the murine CCL2 gene and the comparative position of human ubiquitin promoter (B); sequence is provided in C.

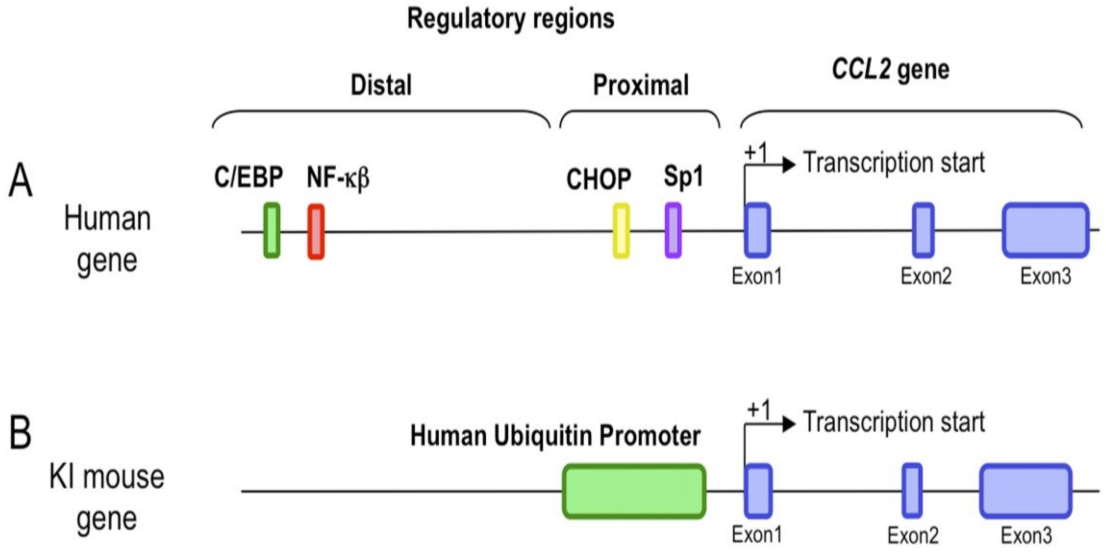

C

```
3661 CAGCAGAAGG ACATTTTAGG ACGGGACTTG GGTGACTCTA GGGCACTGGT TTTCTTTCCA
3721 GAGAGCGGAA CAGGCGAGGA AAAGTAGTCC CTTCTCGGCG ATTCTGCGGA GGGATCTCCG
3781 TGGGGCGGTG AACGCCGATG ATTATATAAG GACGCGCCGG GTGTGGCACA GCTAGTTCCG
3841 TCGCAGCCGG GATTTGGGTC GCGGTTCTTG TTTGTGGATC GCTGTGATCG TCACTTGGTG
3901 AGTAGCGGGC TGCTGGGCTG GCCGGGGCTT TCGTGGCCGC CGGGCCGCTC GGTGGGACGG
3961 AAGCGTGTGG AGAGACCGCC AAGGGCTGTA GTCTGGGTCC GCGAGCAAGG TTGCCCTGAA
4021 CTGGGGGTTG GGGGGAGCGC AGCAAAATGG CGGCTGTTCC CGAGTCTTGA ATGGAAGACG
4081 CTTGTGAGGC GGGCTGTGAG GTCGTTGAAA CAAGGTGGGG GGCATGGTGG GCGGCAAGAA
4141 CCCAAGGTCT TGAGGCCTTC GCTAATGCGG GAAAGCTCTT ATTCGGGTGA GATGGGCTGG
4201 GGCACCATCT GGGGACCCTG ACGTGAAGTT TGTCACTGAC TGGAGAACTC GGTTTGTCGT
4261 CTGTTGCGGG GGCGGCAGTT ATGGCGGTGC CGTTGGGCAG TGCACCCGTA CCTTTGGGAG
4321 CGCGCGCCCT CGTCGTGTCG TGACGTCACC CGTTCTGTTG GCTTATAATG CAGGGTGGGG
4381 CCACCTGCCG GTAGGTGTGC GGTAGGCTTT TCTCCGTCGC AGGACGCAGG GTTCGGGCCT
4441 AGGGTAGGCT CTCCTGAATC GACAGGCGCC GGACCTCTGG TGAGGGGAGG GATAAGTGAG
4501 GCGTCAGTTT CTTTGGTCGG TTTTATGTAC CTATCTTCTT AAGTAGCTGA AGCTCCGGTT
4561 TTGAACTATG CGCTCGGGGT TGGCGAGTGT GTTTTGTGAA GTTTTTTAGG CACCTTTTGA
4621 AATGTAATCA TTTGGGTCAA TATGTAATTT TCAGTGTTAG ACTAGTAAAT TGTCCGCTAA
4681 ATTCTGGCCG TTTTTGGCTT TTTTGTTAGA CGCCACCATG CATATAACTT CGTATAGCAT
```

Supplementary material S2, details of the sequence of other main elements used in the construction of the vector, corresponding to the manuscript:

Ubiquitous transgenic overexpression of C-C chemokine ligand 2: a model to assess the combined effect of high energy intake and continuous low-grade inflammation?

Esther Rodríguez-Gallego, Marta Riera-Borrull, Anna Hernández-Aguilera, Roger Mariné-Casadó, Anna Rull, Raúl Beltrán-Debón, Fedra Luciano-Mateo, Javier A. Menéndez[1], Alejandro Vazquez-Martin[1], Juan J. Sirvent[2], Vicente Martín-Paredero[3], Angel L. Corbí[4], Elena Sierra-Filardi[4], Gerard Aragonès, Anabel García-Heredia, Jordi Camps, Carlos Alonso-Villaverde[5], Jorge Joven*

The sequence of main elements used in the construction of the vector is described according to the following, color-based legend. For clarity, flanking regions were also included.

```
5'homology arm 465-3467
MCS 3475-3495
UbiC promoter 3500-4709
loxP site 4724-4757
STOP cassette 4768-6101
loxP site 6102-6135
genomic fragment of CCL2 gene (frag) 6150-9056
          exon1 6468-6628
          exon2 7374-7491
          exon3 7817-8343
SV40pA 9090-9318
FRT site 9331-9378
PGK promoter 9388-9906
Neo CDS 9974-10777
Splice donor (SD) 10799-11005
Instability signal (IS) 11006-11059
FRT site 11090-11137
MCS 11209-11230
3'homology arm 11231-14260
```

```
  1 AAACCGTCTA TCAGGGCGAT GGCCCACTAC GTGAACCATC ACCCTAATCA AGTTTTTTGG

 61 GGTCGAGGTG CCGTAAAGCA CTAAATCGGA ACCCTAAAGG GAGCCCCCGA TTTAGAGCTT

121 GACGGGGAAA GCCGGCGAAC GTGGCGAGAA AGGAAGGGAA GAAAGCGAAA GGAGCGGGCG

181 CTAGGGCGCT GGCAAGTGTA GCGGTCACGC TGCGCGTAAC CACCACACCC GCCGCGCTTA

241 ATGCGCCGCT ACAGGGCGCG TCCCATTCGC CATTCAGGCT GCGCAACTGT TGGGAAGGGC

301 GATCGGTGCG GGCCTCTTCG CTATTACGCC AGCTGGCGAA AGGGGGATGT GCTGCAAGGC

361 GATTAAGTTG GGTAACGCCA GGGTTTTCCC AGTCACGACG TTGTAAAACG ACGGCCAGTG

421 AATTGTAATA CGACTCACTA TAGGGCGAAT TGGGTACCGG CGCGCCTAGC GACCTAAACC

481 ATTATTAATA TCAAATTAAC CATCAAAACA CTTTCCTCTC AATATGCTGC ACACAAACCT

541 CCTCCTGGAA CCTCCTCCAT CTGGATCCTC CCCAATCAAA AGTATAGGTA TTTAACATAT

601 AAGCAAGGAA GTAATGTAAA CATGACCTTG GTCACAAATA TGTCATCTAA AAACAATTTA
```

661 GTCAAGGTAT GGAGGAAATT CGAGAACCTG AATCTTTTTA AGTATTTTGA GCACAGGAAC

721 AATTGGCAAA AGGAATCCAG GTATAGACAA AACCCAGAGC CCAGAGCTCT GGGCGAAAAA

781 TGAGTTGCTG GTGAAGACGT TACACAAGTA ACATGAGAAA GCAGAAAATG CAGGTCATCC

841 ACGCACCCCT GACCCAGGCC AGCAGGGCGG GCTGCAGCAT CAGTACACAG GAGAAAGATC

901 CTTATTCCTA AGAATGAGAA AGGCAAAGGC GCCCGATAGA ATAAATTAGC ATAGAAGGGG

961 CTTTCCCAGG AGTTAAAACT TTCCTTCTGA GCGATTACCT ACTAAAACCA GGGCTTTTGC

1021 CCACTACCAT TTACCTAGGA TCTTGGCTTG CACGGATTCA TAGGGGCATA TCCCTCCCCC

1081 TCTTCTTTAG AGTCGTTCTT AAAAGATCGC TCTCCACGCC CTAGGCAGGG AAAACGACAA

1141 AATCTGGCTC AATTCCAGGC TAGAACCCTA CAAATTCAAC AGGGATATCG CAAGGATACT

1201 GGGGCATACG CCACAGGGAG TCCAAGAATG TGAGGTGGGG GTGGCGAAGG TAATGTCTTT

1261 GGTGTGGGAA AAGCAGCAGC CATCTGAGAT AGGAACTGGA AAACCAGAGG AGAGGCGTTC

1321 AGGAAGATTA TGGAGGGGAG GACTGGGCCC CCACGAGCGA CCAGAGTTGT CACAAGGCCG

1381 CAAGAACAGG GGAGGTGGGG GGCTCAGGGA CAGAAAAAAA AGTATGTGTA TTTTGAGAGC

1441 AGGGTTGGGA GGCCTCTCCT GAAAAGGGTA TAAACGTGGA GTAGGCAATA CCCAGGCAAA

1501 AAGGGGAGAC CAGAGTAGGG GGAGGGGAAG AGTCCTGACC CAGGGAAGAC ATTAAAAAGG

1561 TAGTGGGGTC GACTAGATGA AGGAGAGCCT TTCTCTCTGG GCAAGAGCGG TGCAATGGTG

1621 TGTAAAGGTA GCTGAGAAGA CGAAAAGGGC AAGCATCTTC CTGCTACCAG GCTGGGGAGG

1681 CCCAGGCCCA CGACCCCGAG GAGAGGGAAC GCAGGGAGAC TGAGGTGACC CTTCTTTCCC

1741 CCGGGGCCCG GTCGTGTGGT TCGGTGTCTC TTTTCTGTTG GACCCTTACC TTGACCCAGG

1801 CGCTGCCGGG GCCTGGGCCC GGGCTGCGGC GCACGGCACT CCCGGGAGGC AGCGAGACTC

1861 GAGTTAGGCC CAACGCGGCG CCACGGCGTT TCCTGGCCGG GAATGGCCCG TACCCGTGAG

1921 GTGGGGGTGG GGGGCAGAAA AGGCGGAGCG AGCCCGAGGC GGGGAGGGGG AGGGCCAGGG

1981 GCGGAGGGGG CCGGCACTAC TGTGTTGGCG GACTGGCGGG ACTAGGGCTG CGTGAGTCTC

2041 TGAGCGCAGC GGGCGGCGGC CGCCCCTCCC CCGGCGGCGG CAGCGGCGGC AGCGGCGGCA

2101 GCTCACTCAG CCCGCTGCCC GAGCGGAAAC GCCACTGACC GCACGGGGAT TCCCAGTGCC

2161 GGCGCCAGGG GCACGCGGGA CACGCCCCCT CCCGCCGCGC CATTGGCCTC TCCGCCCACC

2221 GCCCCACACT TATTGGCCGG TGCGCCGCCA ATCAGCGGAG GCTGCCGGGG CCGCCTAAAG

2281 AAGAGGCTGT GCTTTGGGGC TCCGGCTCCT CAGAGAGCCT CGGCTAGGTA GGGGATCGGG

2341 ACTCTGGCGG GAGGGCGGCT TGGTGCGTTT GCGGGGATGG GCGGCCGCGG CAGGCCCTCC

2401 GAGCGTGGTG GAGCCGTTCT GTGAGACAGC CGGGTACGAG TCGTGACGCT GGAAGGGGCA

2461 AGCGGGTGGT GGGCAGGAAT GCGGTCCGCC CTGCAGCAAC CGGAGGGGGA GGGAGAAGGG

2521 AGCGGAAAAG TCTCCACCGG ACGCGGCCAT GGCTCGGGGG GGGGGGGCA GCGGAGGAGC

2581 GCTTCCGGCC GACGTCTCGT CGCTGATTGG CTTCTTTTCC TCCCGCCGTG TGTGAAAACA

2641 CAAATGGCGT GTTTTGGTTG GCGTAAGGCG CCTGTCAGTT AACGGCAGCC GGAGTGCGCA

2701 GCCGCCGGCA GCCTCGCTCT GCCCACTGGG TGGGGCGGGA GGTAGGTGGG GTGAGGCGAG

2761 CTGGACGTGC GGGCGCGGTC GGCCTCTGGC GGGGCGGGGG AGGGGAGGGA GGGTCAGCGA

2821 AAGTAGCTCG CGCGCGAGCG GCCGCCCACC CTCCCCTTCC TCTGGGGGAG TCGTTTTACC

2881 CGCCGCCGGC CGGGCCTCGT CGTCTGATTG GCTCTCGGGG CCCAGAAAAC TGGCCCTTGC

2941 CATTGGCTCG TGTTCGTGCA AGTTGAGTCC ATCCGCCGGC CAGCGGGGGC GGCGAGGAGG

3001 CGCTCCCAGG TTCCGGCCCT CCCCTCGGCC CCGCGCCGCA GAGTCTGGCC GCGCGCCCCT

3061 GCGCAACGTG GCAGGAAGCG CGCGCTGGGG GCGGGGACGG GCAGTAGGGC TGAGCGGCTG

3121 CGGGGCGGGT GCAAGCACGT TTCCGACTTG AGTTGCCTCA AGAGGGGCGT GCTGAGCCAG

3181 ACCTCCATCG CGCACTCCGG GGAGTGGAGG GAAGGAGCGA GGGCTCAGTT GGGCTGTTTT

3241 GGAGGCAGGA AGCACTTGCT CTCCCAAAGT CGCTCTGAGT TGTTATCAGT AAGGGAGCTG

3301 CAGTGGAGTA GGCGGGGAGA AGGCCGCACC CTTCTCCGGA GGGGGGAGGG GAGTGTTGCA

3361 ATACCTTTCT GGGAGTTCTC TGCTGCCTCC TGGCTTCTGA GGACCGCCCT GGGCCTGGGA

3421 GAATCCCTTC CCCCTCTTCC CTCGTGATCT GCAACTCCAG TCTTTCTGGT ACCT**CTAGAC**

3481**ATATGTTAAT TAAAT**CGATG GCCTCCGCGC CGGGTTTTGG CGCCTCCCGC GGGCGCCCCC

3541 CTCCTCACGG CGAGCGCTGC CACGTCAGAC GAAGGGCGCA GCGAGCGTCC TGATCCTTCC

3601 GCCCGGACGC TCAGGACAGC GGCCCGCTGC TCATAAGACT CGGCCTTAGA ACCCCAGTAT

3661 CAGCAGAAGG ACATTTTAGG ACGGGACTTG GGTGACTCTA GGGCACTGGT TTTCTTTCCA

3721 GAGAGCGGAA CAGGCGAGGA AAAGTAGTCC CTTCTCGGCG ATTCTGCGGA GGGATCTCCG

3781 TGGGGCGGTG AACGCCGATG ATTATATAAG GACGCGCCGG GTGTGGCACA GCTAGTTCCG

3841 TCGCAGCCGG GATTTGGGTC GCGGTTCTTG TTTGTGGATC GCTGTGATCG TCACTTGGTG

3901 AGTAGCGGGC TGCTGGGCTG GCCGGGGCTT TCGTGGCCGC CGGGCCGCTC GGTGGGACGG

3961 AAGCGTGTGG AGAGACCGCC AAGGGCTGTA GTCTGGGTCC GCGAGCAAGG TTGCCCTGAA

4021 CTGGGGGTTG GGGGGAGCGC AGCAAAATGG CGGCTGTTCC CGAGTCTTGA ATGGAAGACG

4081 CTTGTGAGGC GGGCTGTGAG GTCGTTGAAA CAAGGTGGGG GGCATGGTGG GCGGCAAGAA

4141 CCCAAGGTCT TGAGGCCTTC GCTAATGCGG GAAAGCTCTT ATTCGGGTGA GATGGGCTGG

4201 GGCACCATCT GGGGACCCTG ACGTGAAGTT TGTCACTGAC TGGAGAACTC GGTTTGTCGT

4261 CTGTTGCGGG GGCGGCAGTT ATGGCGGTGC CGTTGGGCAG TGCACCCGTA CCTTTGGGAG

4321 CGCGCGCCCT CGTCGTGTCG TGACGTCACC CGTTCTGTTG GCTTATAATG CAGGGTGGGG

4381 CCACCTGCCG GTAGGTGTGC GGTAGGCTTT TCTCCGTCGC AGGACGCAGG GTTCGGGCCT

4441 AGGGTAGGCT CTCCTGAATC GACAGGCGCC GGACCTCTGG TGAGGGGAGG GATAAGTGAG

4501 GCGTCAGTTT CTTTGGTCGG TTTTATGTAC CTATCTTCTT AAGTAGCTGA AGCTCCGGTT

4561 TTGAACTATG CGCTCGGGGT TGGCGAGTGT GTTTTGTGAA GTTTTTTAGG CACCTTTTGA

4621 AATGTAATCA TTTGGGTCAA TATGTAATTT TCAGTGTTAG ACTAGTAAAT TGTCCGCTAA

4681 ATTCTGGCCG TTTTTGGCTT TTTTGTTAGA CGCCACCATG CATATAACTT CGTATAGCAT

4741 ACATTATACG AAGTTATGGC GCGCCGATCC TCGGGGACAC CAAATATGGC GATCTCGGCC

4801 TTTTCGTTTC TTGGAGCTGG GACATGTTTG CCATCGATCC ATCTACCACC AGAACGGCCG

4861 TTAGATCTGC TGCCACCGTT GTTTCCACCG AAGAAACCAC CGTTGCCGTA ACCACCACGA

4921 CGGTTGTTGC TAAAGAAGCT GCCACCGCCA CGGCCACCGT TGTAGCCGCC GTTGTTGTTA

4981 TTGTAGCTGC TACTGTTATT TCTGGCACTT CTTGGTTTTC CTCTTAAGTG AGGAGGAACA

5041 TAACCATTCT CGTTGTTGTC GTTGATGCTT AAATTTTGCA CTTGTTCGCT CAGTTCAGCC

5101 ATAATATGAA ATGCTTTTCT TGTTGTTCTT ACGGAATACC ACTTGCCACC TATCACCACA

5161 ACTAACTTTT TCCCGTTCCT CCATCTCTTT TATATTTTTT TTCTCGAGGG ATCTTTGTGA

5221 AGGAACCTTA CTTCTGTGGT GTGACATAAT TGGACAAACT ACCTACAGAG ATTTAAAGCT

5281 CTAAGGTAAA TATAAAATTT TTAAGTGTAT AATGTGTTAA ACTACTGATT CTAATTGTTT

5341 GTGTATTTTA GATTCCAACC TATGGAACTG ATGAATGGGA GCAGTGGTGG AATGCCTTTA

5401 ATGAGGAAAA CCTGTTTTGC TCAGAAGAAA TGCCATCTAG TGATGATGAG GCTACTGCTG

5461 ACTCTCAACA TTCTACTCCT CCAAAAAAGA AGAGAAAGGT AGAAGACCCC AAGGACTTTC

5521 CTTCAGAATT GCTAAGTTTT TTGAGTCATG CTGTGTTTAG TAATAGAACT CTTGCTTGCT

5581 TTGCTATTTA CACCACAAAG GAAAAAGCTG CACTGCTATA CAAGAAAATT ATGGAAAAAT

5641 ATTCTGTAAC CTTTATAAGT AGGCATAACA GTTATAATCA TAACATACTG TTTTTTCTTA

5701 CTCCACACAG GCATAGAGTG TCTGCTATTA ATAACTATGC TCAAAAAATTG TGTACCTTTA

5761 GCTTTTTAAT TTGTAAAGGG GTTAATAAGG AATATTTGAT GTATAGTGCC TTGACTAGAG

5821 ATCATAATCA GCCATACCAC ATTTGTAGAG GTTTTACTTG CTTTAAAAAA CCTCCCACAC

5881 CTCCCCCTGA ACCTGAAACA TAAAATGAAT GCAATTGTTG TTGTTAACTT GTTTATTGCA

5941 GCTTATAATG GTTACAAATA AAGCAATAGC ATCACAAATT TCACAAATAA AGCATTTTTT

6001 TCACTGCATT CTAGTTGTGG TTTGTCCAAA CTCATCAATG TATCTTATCA TGTCTGGATC

6061 TGACATGGTA AGTAAGCTTG GGCTGCAGGT CGAGGGACCT AATAACTTCG TATAGCATAC

6121 ATTATACGAA GTTATGCGGC CGCGAATTCA AAGCAGAGCC ACTCCATTCA CACTTGTGGT

6181 CACAGTAGTA CAATTACTGC CAATTCTTCC CTCTTTCCCC CCCCCCCCCC CTACTCCCTG

6241 CGCAGCTTCA TTTGCTCCCA GGAGTGGCTA GAAAAATACC AAATTCCAAC CCACAGTTTC

6301 TCTCTTCCAC TTCCTGGAAA CACCCGAGGG CTCTGCACTT ACTCAGCGGA TTCAACTTCC

6361 ACTTTCCATC ACTTATCCAG GGTGATGCTA CTCCTTGGCA CCAAGCACCC TGCCTGACTC

6421 CACCCCCCTG GCTTACAATA AAAGGCTGCC TCAGAGCAGC CAGAAGTGCA GAGAGCCAGA

6481 CGGGAGGAAG GCCAGCCCAG CACCAGCACC AGCCAACTCT CACTGAAGCC AGCTCTCTCT

6541 TCCTCCACCA CCATGCAGGT CCCTGTCATG CTTCTGGGCC TGCTGTTCAC AGTTGCCGGC

6601 TGGAGCATCC ACGTGTTGGC TCAGCCAGGT GAGACCCCCC CAGCTCCCTT CCCAGCATAT

6661 CACCCCATTT TTGAATTGTC GTGGATTGTG ATAGCATAGT CTCACATGGT CAGGTACTTT

6721 TTTTTTTTTC TTTAACCAAG ATAAGGAGCA TAAAGAAGGA AGGACAAAGA GCCAACCCAA

6781 TTACAAGATT GCTTCTGGAA AGCAACTAGA ATTTTAATTG TTAGATCTAA ATTTGGAATC

6841 ACACCTTCAT ATAGTTCCTG TTCCAGTTAC TTCCCTCAGT ATTTGGGAAC CTGGGTGATC

6901 AAACAGAGGC TTGGGTTGGT GCCTTTTTCC AGATAGAGGA GAAAGGGGAA GAGATCCAAG

6961 ATCCGAGCTG TGTTTCACCC AGCCCTGCTT CCAGAGATAG CAGCTTAGCG GAGGTGGTTG

7021 GGATCAGAGA TACTCATGAT TTGATTTTTT TTTTTTTTTT TTTTTTTTTT TTTTTTTTGC

7081 AGCCTACAGT AATGTACTCA GGTAATCTTC TCAGGTCATA GTAATTTGAC TTCTAACTCC

7141 CCCAAATGAC AGTCCCCAGA GTCACATAGT TTTAATGGCA TCCCTCTACC CAAGACTGTG

7201 AGCCTACTTT AAGCTGCAAA TAACTGAGTC TGTTGTCAAA GATCACATCC CAGATCTGAT

7261 GTATTGGCAT TTATATCCCA TCCTGCTGAA ACTGCCTTCT CCCCGTGGTC CTTCTCTTCT

7321 CTAAGGTCAG AAGCATCTTT CCTGTCCTAA TGTGCTTCTC TTTTACTCTC CAGATGCAGT

7381 TAACGCCCCA CTCACCTGCT GCTACTCATT CACCAGCAAG ATGATCCCAA TGAGTAGGCT

7441 GGAGAGCTAC AAGAGGATCA CCAGCAGCAG GTGTCCCAAA GAAGCTGTAG TGTGAGTTAC

7501 ATACCCCGGC CCTCCCTGGT CCAAAGGTTT TTCCTTAAGA ACAAGGGATG GTCCTCATAT

7561 ACTTATAGTC AGTCACACAC TCAGATCCAA TGGGGAAACC AAGGCCAAGA AGGCAAAGGC

7621 AGTTCTCAAC AGCATTGTCT CTATGGCTGC TGTTCAGGCC CTTTCTACTC CACAAGCTTA

7681 TCTTAGAAAA CCTGCAGGAG AAGCAGGTCA CTTTGAGTCC CCTTTTTCTA CCTGCCCTCC

7741 CCCGCTGAGC TCTACACAGC CCCTCCATGT ATACCAGACT GAACTTCATC TAACAGTGTC

7801 TTTTCTCTTC CCACAGTTTT GTCACCAAGC TCAAGAGAGA GGTCTGTGCT GACCCCAAGA

7861 AGGAATGGGT CCAGACATAC ATTAAAAACC TGGATCGGAA CCAAATGAGA TCAGAACCTA

7921 CAACTTTATT TAAAACTGCA TCTGCCCTAA GGTCTTCAGC ACCTTTGAAT GTGAAGTTGA

7981 CCCGTAAATC TGAAGCTAAT GCATCCACTA CCTTTTCCAC AACCACCTCA AGCACTTCTG

8041 TAGGAGTGAC CAGTGTGACA GTGAACTAGT GTGACTCGGA CTGTGATGCC TTAATTAATA

8101 TTAAACTTAT TTAACTTATT GATGTTATGG TATTCCCTTT CATGAATACT AAAATTTCTT

8161 AAATGCAAGG TGTGGATCCA TTTTTCCCTC TCTGTGAATC CAGATTCAAC ACTTTCAATG

8221 TATGAGAGAT GAATTTTGTA AAGATGAATG GGTAAACTTT GTGTTTGAGA TTCCAAGGTA

8281 TTGTTTAAAA TATTATTATG GATATTCCTT ATTATTAAAA GAAATATATT ATTTTTGTAC

8341 ACCAGTCTGA CTTTGAGTGT TTTCTTGAGG GAAACTGCAA AGCTGAGAGT ATATAAGCTT

8401 GGAGAGCAAC ACAGGTTGGG ACACATATTG TGGGGGAACA GGAGAGTGAA TGGCCCACTC

8461 TTTTGTATTG AATGGTCTTA TCTGAGTGTC ATAGACTCTT CAAGATGGGG CCCAGTCAGG

8521 GATGCTAGTA CCATTGTTTT GGTCCCTAGC ATTGCTTCTC AGATACATGC TCAACAAAAG

8581 CCCCAGTCCT TCCCAGTCAC ATGTCCTAGT TGCCCTTTAA CTGGGATACA TCACTTCCAT

8641 CTACAGCTCT GCAATTTATA ATTCTCTAAC TGCATAGGTA GTTCAGACAG TGGAGAACAG

8701 ACGGGAGACT TAACCATCTT CTATAGGAAG GTTATGGTAA TGCTTCCTAG AGAGAGGAAG

8761 ACATGAACTT ATCCTTGATA ATGGTTAGTT TTATAGGAAA AAAAACACAA AAGCATTAAG

8821 GAAAACTGGT AGGTGGGGGA AATTATCCAA AAATAGAAAT GAAAATGGTG TGTACATTAA

8881 ACGGGGAAGA GCGAGGGAAA CCATCCGATG GAGCTGCATG TATATCAAGA GATGGGGATT

8941 TTTCAAGAGG AAGCTCAATA CAGGGTTTGG AATAGGAAGG TGAGAACATG GTATTCCCTC

9001 GGACCTATAA TTTAAAGGAT AAGTTGAAAC TCTTCGGTCC AATAAAGAGG GAGGGAGAAT

9061 TCAGATCTGG TACCGATATC AAGCTTAACT GATCATAATC AGCCATACCA CATTTGTAGA

9121 GGTTTTACTT GCTTTAAAAA ACCTCCCACA CCTCCCCCTG AACCTGAAAC ATAAAATGAA

9181 TGCAATTGTT GTTGTTAACT TGTTTATTGC AGCTTATAAT GGTTACAAAT AAAGCAATAG

9241 CATCACAAAT TTCACAAATA AAGCATTTTT TTCACTGCAT TCTAGTTGTG GTTTGTCCAA

9301 ACTCATCAAT GTATCTTAAC GCTTAATTAA GAAGTTCCTA TTCCGAAGTT CCTATTCTCT

9361 AGTAAGTATA GGAACTTCTG GCGAATTAAT TCTACCGGGT AGGGGAGGCG CTTTTCCCAA

9421 GGCAGTCTGG AGCATGCGCT TTAGCAGCCC CGCTGGGCAC TTGGCGCTAC ACAAGTGGCC

9481 TCTGGCCTCG CACACATTCC ACATCCACCG GTAGGCGCCA ACCGGCTCCG TTCTTTGGTG

9541 GCCCCTTCGC GCCACCTTCT ACTCCTCCCC TAGTCAGGAA GTTCCCCCCC GCCCCGCAGC

9601 TCGCGTCGTG CAGGACGTGA CAAATGGAAG TAGCACGTCT CACTAGTCTC GTGCAGATGG

9661 ACAGCACCGC TGAGCAATGG AAGCGGGTAG GCCTTTGGGG CAGCGGCCAA TAGCAGCTTT

9721 GCTCCTTCGC TTTCTGGGCT CAGAGGCTGG GAAGGGGTGG GTCCGGGGGC GGGCTCAGGG

9781 GCGGGCTCAG GGGCGGGGCG GGCGCCCGAA GGTCCTCCGG AGGCCCGGCA TTCTGCACGC

9841 TTCAAAAGCG CACGTCTGCC GCGCTGTTCT CCTCTTCCTC ATCTCCGGGC CTTTCGACCT

9901 GCAGCCTGTT GACAATTAAT CATCGGCATA GTATATCGGC ATAGTATAAT ACGACAAGGT

9961 GAGGAACTAA ACCATGGGAT CGGCCATTGA ACAAGATGGA TTGCACGCAG GTTCTCCGGC

10021 CGCTTGGGTG GAGAGGCTAT TCGGCTATGA CTGGGCACAA CAGACAATCG GCTGCTCTGA

10081 TGCCGCCGTG TTCCGGCTGT CAGCGCAGGG GCGCCCGGTT CTTTTTGTCA AGACCGACCT

10141 GTCCGGTGCC CTGAATGAAC TGCAGGACGA GGCAGCGCGG CTATCGTGGC TGGCCACGAC

10201 GGGCGTTCCT TGCGCAGCTG TGCTCGACGT TGTCACTGAA GCGGGAAGGG ACTGGCTGCT

10261 ATTGGGCGAA GTGCCGGGGC AGGATCTCCT GTCATCTCAC CTTGCTCCTG CCGAGAAAGT

10321 ATCCATCATG GCTGATGCAA TGCGGCGGCT GCATACGCTT GATCCGGCTA CCTGCCCATT

10381 CGACCACCAA GCGAAACATC GCATCGAGCG AGCACGTACT CGGATGGAAG CCGGTCTTGT

10441 CGATCAGGAT GATCTGGACG AAGAGCATCA GGGGCTCGCG CCAGCCGAAC TGTTCGCCAG

10501 GCTCAAGGCG CGCATGCCCG ACGGCGATGA TCTCGTCGTG ACCCATGGCG ATGCCTGCTT

10561 GCCGAATATC ATGGTGGAAA ATGGCCGCTT TTCTGGATTC ATCGACTGTG GCCGGCTGGG

10621 TGTGGCGGAC CGCTATCAGG ACATAGCGTT GGCTACCCGT GATATTGCTG AAGAGCTTGG

10681 CGGCGAATGG GCTGACCGCT TCCTCGTGCT TTACGGTATC GCCGCTCCCG ATTCGCAGCG

10741 CATCGCCTTC TATCGCCTTC TTGACGAGTT CTTCTGAGGC CGGCCCCACG CGTCCACCTT

10801 TGTTGTTGGA TATGCCCTTG ACTATAATGA GTACTTCAGG GATTTGAATG TAAGTAATGC

10861 TTTTTTTTTT CTCGCTCTCA TTTTTCAAAA ACCCACATAA AAATTGAGGA AAGGGAAGAA

10921 TTGTTTTCTC CTTCCAGCAC CTCGTAATTT GACCCGACTG ATGGTTCCCA TTAGTCACAT

10981 AAAGCTGTAG TCAAGTACAG ACGTCAGTAA TATTTATATA TTTATATTTT TAAAATATTT

11041 ATTTATTTAT TTATTTAAGT TAATTAGTGA AGGGCGAGCT TGAGGATCTG AAGTTCCTAT

11101 TCCGAAGTTC CTATTCTCTA GTAAGTATAG GAACTTCGGA TCGTTGAAGA AGGAGGTGGG

11161 ATGCTTAATT AAGCTTGTCG ACTCTAGATT GCGGCCGCGG CGCGCCAT**CG ATGAATTCGA**

11221 **TATCGGCCGG**CCGAAGATGG GCGGGAGTCT TCTGGGCAGG CTTAAAGGCT AACCTGGTGT

11281 GTGGGCGTTG TCCTGCAGGG GAATTGAACA GGTGTAAAAT TGGAGGGACA AGACTTCCCA

11341 CAGATTTTCG GTTTTGTCGG GAAGTTTTTT AATAGGGGCA AATAAGGAAA ATGGGAGGAT

11401 AGGTAGTCAT CTGGGGTTTT ATGCAGCAAA ACTACAGGTT ATTATTGCTT GTGATCCGCC

11461 TCGGAGTATT TTCCATCGAG GTAGATTAAA GACATGCTCA CCCGAGTTTT ATACTCTCCT

11521 GCTTGAGATC CTTACTACAG TATGAAATTA CAGTGTCGCG AGTTAGACTA TGTAAGCAGA

11581 ATTTTAATCA TTTTTAAAGA GCCCAGTACT TCATATCCAT TTCTCCCGCT CCTTCTGCAG

11641 CCTTATCAAA AGGTATTTTA GAACACTCAT TTTAGCCCCA TTTTCATTTA TTATACTGGC

11701 TTATCCAACC CCTAGACAGA GCATTGGCAT TTTCCCTTTC CTGATCTTAG AAGTCTGATG

11761 ACTCATGAAA CCAGACAGAT TAGTTACATA CACCACAAAT CGAGGCTGTA GCTGGGGCCT

11821 CAACACTGCA GTTCTTTTAT AACTCCTTAG TACACTTTTT GTTGATCCTT TGCCTTGATC

11881 CTTAATTTTC AGTGTCTATC ACCTCTCCCG TCAGGTGGTG TTCCACATTT GGGCCTATTC

11941 TCAGTCCAGG GAGTTTTACA ACAATAGATG TATTGAGAAT CCAACCTAAA GCTTAACTTT

12001 CCACTCCCAT GAATGCCTCT CTCCTTTTTC TCCATTTATA AACTGAGCTA TTAACCATTA

12061 ATGGTTTCCA GGTGGATGTC TCCTCCCCCA ATATTACCTG ATGTATCTTA CATATTGCCA

12121 GGCTGATATT TTAAGACATT AAAAGGTATA TTTCATTATT GAGCCACATG GTATTGATTA

12181 CTGCTTACTA AAATTTTGTC ATTGTACACA TCTGTAAAAG GTGGTTCCTT TTGGAATGCA

12241 AAGTTCAGGT GTTTGTTGTC TTTCCTGACC TAAGGTCTTG TGAGCTTGTA TTTTTTCTAT

12301 TTAAGCAGTG CTTTCTCTTG GACTGGCTTG ACTCATGGCA TTCTACACGT TATTGCTGGT

12361 CTAAATGTGA TTTTGCCAAG CTTCTTCAGG ACCTATAATT TTGCTTGACT TGTAGCCAAA

12421 CACAAGTAAA ATGATTAAGC AACAAATGTA TTTGTGAAGC TTGGTTTTTA GGTTGTTGTG

12481 TTGTGTGTGC TTGTGCTCTA TAATAATACT ATCCAGGGGC TGGAGAGGTG GCTCGGAGTT

12541 CAAGAGCACA GACTGCTCTT CCAGAAGTCC TGAGTTCAAT TCCCAGCAAC CACATGGTGG

12601 CTCACAACCA TCTGTAATGG GATCTGATGC CCTCTTCTGG TGTGTCTGAA GACCACAAGT

12661 GTATTCACAT TAAATAAATA AATCCTCCTT CTTCTTCTTT TTTTTTTTTT TAAAGAGAAT

12721 ACTGTCTCCA GTAGAATTTA CTGAAGTAAT GAAATACTTT GTGTTTGTTC CAATATGGTA

12781 GCCAATAATC AAATTACTCT TTAAGCACTG GAAATGTTAC CAAGGAACTA ATTTTTATTT

12841 GAAGTGTAAC TGTGGACAGA GGAGCCATAA CTGCAGACTT GTGGGATACA GAAGACCAAT

12901 GCAGACTTTA ATGTCTTTTC TCTTACACTA AGCAATAAAG AAATAAAAAT TGAACTTCTA

12961 GTATCCTATT TGTTTAAACT GCTAGCTTTA CTTAACTTTT GTGCTTCATC TATACAAAGC

13021 TGAAAGCTAA GTCTGCAGCC ATTACTAAAC ATGAAAGCAA GTAATGATAA TTTTGGATTT

13081 CAAAAATGTA GGGCCAGAGT TTAGCCAGCC AGTGGTGGTG CTTGCCTTTA TGCCTTTAAT

13141 CCCAGCACTC TGGAGGCAGA GACAGGCAGA TCTCTGAGTT TGAGCCCAGC CTGGTCTACA

13201 CATCAAGTTC TATCTAGGAT AGCCAGGAAT ACACACAGAA ACCCTGTTGG GGAGGGGGGC

13261 TCTGAGATTT CATAAAATTA TAATTGAAGC ATTCCCTAAT GAGCCACTAT GGATGTGGCT

13321 AAATCCGTCT ACCTTTCTGA TGAGATTTGG GTATTATTTT TTCTGTCTCT GCTGTTGGTT

13381 GGGTCTTTTG ACACTGTGGG CTTTCTTTAA AGCCTCCTTC CTGCCATGTG GTCTCTTGTT

13441 TGCTACTAAC TTCCCATGGC TTAAATGGCA TGGCTTTTTG CCTTCTAAGG GCAGCTGCTG

13501 AGATTTGCAG CCTGATTTCC AGGGTGGGGT TGGGAAATCT TTCAAACACT AAAATTGTCC

13561 TTTAATTTTT TTTTTAAAAA ATGGGTTATA TAATAAACCT CATAAAATAG TTATGAGGAG

13621 TGAGGTGGAC TAATATTAAA TGAGTCCCTC CCCTATAAAA GAGCTATTAA GGCTTTTTGT

13681 CTTATACTTA ACTTTTTTTT TAAATGTGGT ATCTTTAGAA CCAAGGGTCT TAGAGTTTTA

13741 GTATACAGAA ACTGTTGCAT CGCTTAATCA GATTTTCTAG TTTCAAATCC AGAGAATCCA

13801 AATTCTTCAC AGCCAAAGTC AAATTAAGAA TTTCTGACTT TTAATGTTAA TTTGCTTACT

13861 GTGAATATAA AAATGATAGC TTTTCCTGAG GCAGGGTCTC ACTATGTATC TCTGCCTGAT

13921 CTGCAACAAG ATATGTAGAC TAAAGTTCTG CCTGCTTTTG TCTCCTGAAT ACTAAGGTTA

13981 AAATGTAGTA ATACTTTTGG AACTTGCAGG TCAGATTCTT TTATAGGGGA CACACTAAGG

14041 GAGCTTGGGT GATAGTTGGT AAAATGTGTT TCAAGTGATG AAAACTTGAA TTATTATCAC

14101 CGCAACCTAC TTTTTAAAAA AAAAAGCCAG GCCTGTTAGA GCATGCTTAA GGGATCCCTA

14161 GGACTTGCTG AGCACACAAG AGTAGTTACT TGGCAGGCTC CTGGTGAGAG CATATTTCAA

14221 AAAACAAGGC AGACAACCAA GAAACTACAG TTAAGGCCGG CCGCGGTGGA GCTCCAGCTT

14281 TTGTTCCCTT TAGTGAGGGT TAATTTCGAG CTTGGCGTAA TCATGGTCAT AGCTGTTTCC

14341 TGTGTGAAAT TGTTATCCGC TCACAATTCC ACACAACATA CGAGCCGGAA GCATAAAGTG

14401 TAAAGCCTGG GGTGCCTAAT GAGTGAGCTA ACTCACATTA ATTGCGTTGC GCTCACTGCC

14461 CGCTTTCCAG TCGGGAAACC TGTCGTGCCA GCTGCATTAA TGAATCGGCC AACGCGCGGG

14521 GAGAGGCGGT TTGCGTATTG GGCGCTCTTC CGCTTCCTCG CTCACTGACT CGCTGCGCTC

14581 GGTCGTTCGG CTGCGGCGAG CGGTATCAGC TCACTCAAAG GCGGTAATAC GGTTATCCAC

14641 AGAATCAGGG GATAACGCAG GAAAGAACAT GTGAGCAAAA GGCCAGCAAA AGGCCAGGAA

14701 CCGTAAAAAG GCCGCGTTGC TGGCGTTTTT CCATAGGCTC CGCCCCCCTG ACGAGCATCA

14761 CAAAAATCGA CGCTCAAGTC AGAGGTGGCG AAACCCGACA GGACTATAAA GATACCAGGC

14821 GTTTCCCCCT GGAAGCTCCC TCGTGCGCTC TCCTGTTCCG ACCCTGCCGC TTACCGGATA

14881 CCTGTCCGCC TTTCTCCCTT CGGGAAGCGT GGCGCTTTCT CATAGCTCAC GCTGTAGGTA

14941 TCTCAGTTCG GTGTAGGTCG TTCGCTCCAA GCTGGGCTGT GTGCACGAAC CCCCCGTTCA

15001 GCCCGACCGC TGCGCCTTAT CCGGTAACTA TCGTCTTGAG TCCAACCCGG TAAGACACGA

15061 CTTATCGCCA CTGGCAGCAG CCACTGGTAA CAGGATTAGC AGAGCGAGGT ATGTAGGCGG

15121 TGCTACAGAG TTCTTGAAGT GGTGGCCTAA CTACGGCTAC ACTAGAAGGA CAGTATTTGG

15181 TATCTGCGCT CTGCTGAAGC CAGTTACCTT CGGAAAAAGA GTTGGTAGCT CTTGATCCGG

15241 CAAACAAACC ACCGCTGGTA GCGGTGGTTT TTTTGTTTGC AAGCAGCAGA TTACGCGCAG

15301 AAAAAAAGGA TCTCAAGAAG ATCCTTTGAT CTTTTCTACG GGGTCTGACG CTCAGTGGAA

15361 CGAAAACTCA CGTTAAGGGA TTTTGGTCAT GAGATTATCA AAAAGGATCT TCACCTAGAT

15421 CCTTTTAAAT TAAAAATGAA GTTTTAAATC AATCTAAAGT ATATATGAGT AAACTTGGTC

15481 TGACAGTTAC CAATGCTTAA TCAGTGAGGC ACCTATCTCA GCGATCTGTC TATTTCGTTC

15541 ATCCATAGTT GCCTGACTCC CCGTCGTGTA GATAACTACG ATACGGGAGG GCTTACCATC

15601 TGGCCCCAGT GCTGCAATGA TACCGCGAGA CCCACGCTCA CCGGCTCCAG ATTTATCAGC

15661 AATAAACCAG CCAGCCGGAA GGGCCGAGCG CAGAAGTGGT CCTGCAACTT TATCCGCCTC

15721 CATCCAGTCT ATTAATTGTT GCCGGGAAGC TAGAGTAAGT AGTTCGCCAG TTAATAGTTT

15781 GCGCAACGTT GTTGCCATTG CTACAGGCAT CGTGGTGTCA CGCTCGTCGT TTGGTATGGC

15841 TTCATTCAGC TCCGGTTCCC AACGATCAAG GCGAGTTACA TGATCCCCCA TGTTGTGCAA

```
15901 AAAAGCGGTT AGCTCCTTCG GTCCTCCGAT CGTTGTCAGA AGTAAGTTGG CCGCAGTGTT

15961 ATCACTCATG GTTATGGCAG CACTGCATAA TTCTCTTACT GTCATGCCAT CCGTAAGATG

16021 CTTTTCTGTG ACTGGTGAGT ACTCAACCAA GTCATTCTGA GAATAGTGTA TGCGGCGACC

16081 GAGTTGCTCT TGCCCGGCGT CAATACGGGA TAATACCGCG CCACATAGCA GAACTTTAAA

16141 AGTGCTCATC ATTGGAAAAC GTTCTTCGGG GCGAAAACTC TCAAGGATCT TACCGCTGTT

16201 GAGATCCAGT TCGATGTAAC CCACTCGTGC ACCCAACTGA TCTTCAGCAT CTTTTACTTT

16261 CACCAGCGTT TCTGGGTGAG CAAAAACAGG AAGGCAAAAT GCCGCAAAAA AGGGAATAAG

16321 GGCGACACGG AAATGTTGAA TACTCATACT CTTCCTTTTT CAATATTATT GAAGCATTTA

16381 TCAGGGTTAT TGTCTCATGA GCGGATACAT ATTTGAATGT ATTTAGAAAA ATAAACAAAT

16441 AGGGGTTCCG CGCACATTTC CCCGAAAAGT GCCACCTAAA TTGTAAGCGT TAATATTTTG

16501 TTAAAATTCG CGTTAAATTT TTGTTAAATC AGCTCATTTT TTAACCAATA GGCCGAAATC

16561 GGCAAAATCC CTGTTTAAAC TCGCGACGCG TTATAAATCA AAAGAATAGA CCGAGATAGG
```

Supplementary material S3, details on the construct design, corresponding to the manuscript:

Ubiquitous transgenic overexpression of C-C chemokine ligand 2: a model to assess the combined effect of high energy intake and continuous low-grade inflammation?

Esther Rodríguez-Gallego, Marta Riera-Borrull, Anna Hernández-Aguilera, Roger Mariné-Casadó, Anna Rull, Raúl Beltrán-Debón, Fedra Luciano-Mateo, Javier A. Menéndez[1], Alejandro Vazquez-Martin[1], Juan J. Sirvent[2], Vicente Martín-Paredero[3], Angel L. Corbí[4], Elena Sierra-Filardi[4], Gerard Aragonès, Anabel García-Heredia, Jordi Camps, Carlos Alonso-Villaverde[5], Jorge Joven*

The Knock-in Construct Design, from plasmid to electroporation

1. Overview

1.1 Vector backbone

The following plasmid (named *Ubi-Neo*) was used for construction of the *targeting vector*.

Ubi-Neo

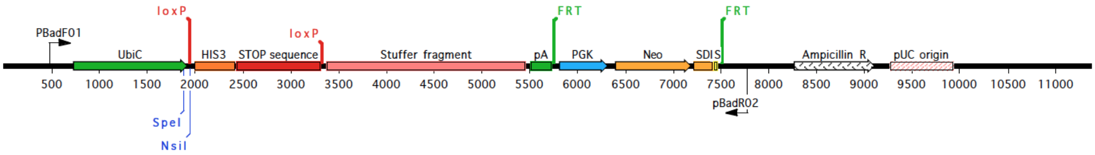

1.2 Construction fragments

Named *frag arm*, contained the genomic fragment of the gene to be introduced (*Ccl2*).

2. *frag arm* (Generation, Diagnostics and Sequencing)

2.1 Production

Using the following primers, the genomic fragment of *Ccl2* gene was amplified by PCR.

Template: C57BL/6 genomic DNA

P_01 5' *AGAATTC*AAAGCAGAGCCACTCCATTCACAC

EcoRI Homology

P_02 5' *TGAATTC*TCCCTCCCTCTTTATTGGACCGAAG

EcoRI Homology

Product: 2921 bp

2.2 Cloning into pPCR

a) Sequencing with PBadF01 or PBadR02

The PCR product was cloned into pPCR and screened by sequencing to confirm clones with the correct insert.

frag arm

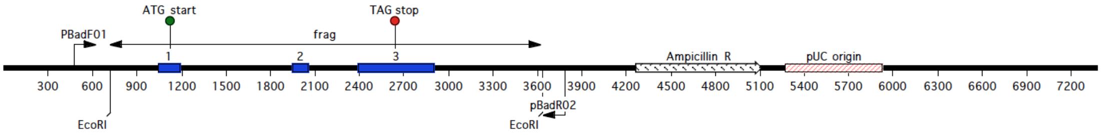

b) Full Sequencing

Once end sequencing identified clones, a full sequence was completed using the following primers.

frag arm

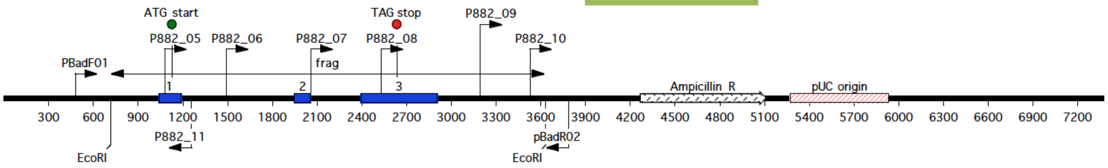

2.3 Cloning into the targeting vector

a) Restriction enzyme digest for cloning

Both *frag arm* vector (containing Ccl2 gene) and the *Ubi-Neo* vector, were digested with EcoRI for cloning *Ccl2* gene into *Ubi-Neo* vector (just where the *Stuffer fragment* was placed).

The resulting vector was named *Ubi-frag-Neo*

frag arm

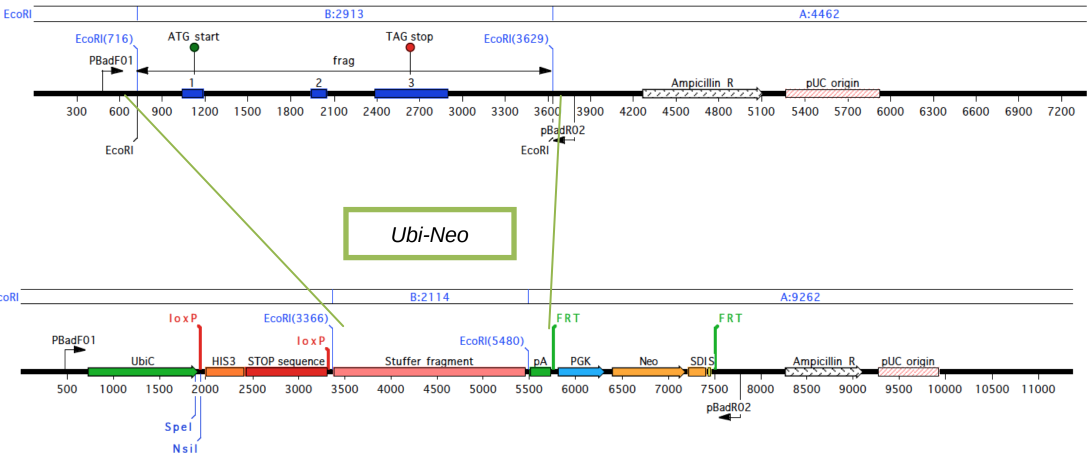

0

b) Screen by Sequencing

Clones containing the insert could be screened by end sequencing with P335_11 and PNeoR10 primers:

Ubi-frag-Neo

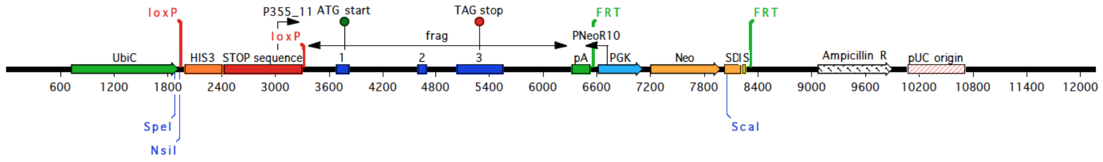

3. Cloning into the ROSA 26 vector

3.1 Cloning into the targeting vector

a) Restriction enzyme digest for cloning

ClaI restriction enzyme was used for cloning the constructed fragment (*Ubi-frag-Neo*) into the *ROSA26 vector* (which contained homologous sequences to ROSA26).

Ubi-frag-Neo

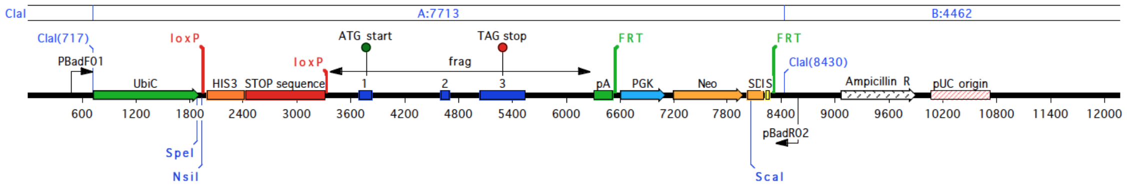

ROSA26 vector

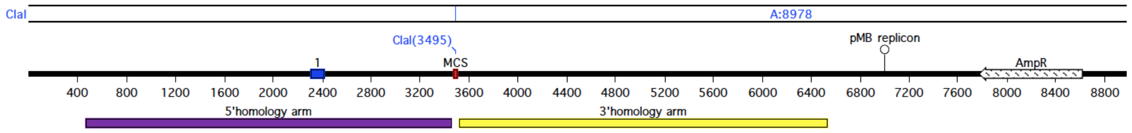

b) Screen by Sequencing

Clones that contained the insert (the *targeting vector*), were screened by end sequencing with the following primers

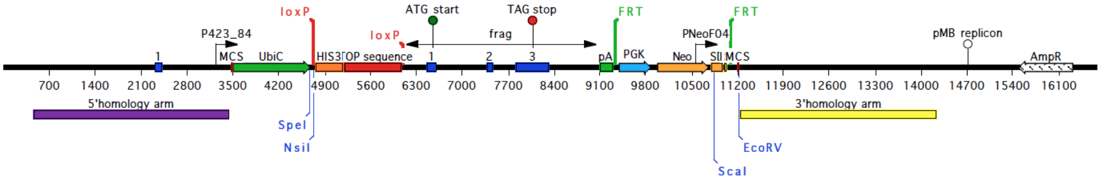

4. Complete *Targeting Vector*

4.1 Final sequencing

To assess the *targeting vector* integrity, final sequencing was performed using the primers identified in the following graphic:

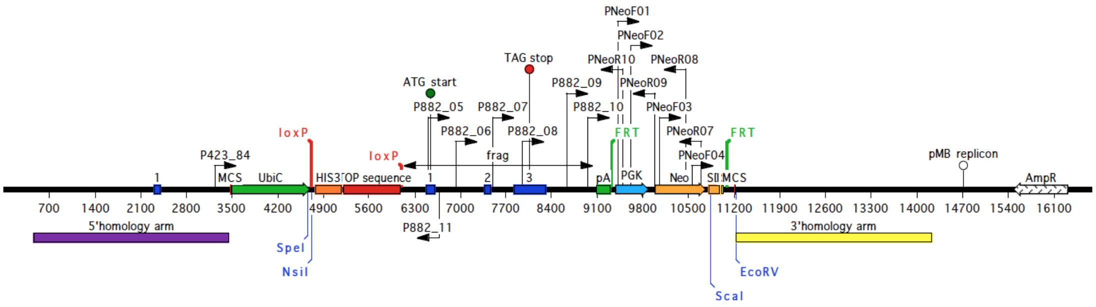

4.2 Restriction enzyme digest for Quality Control

Targeting vector was digested with several restriction enzymes as quality control strategy:

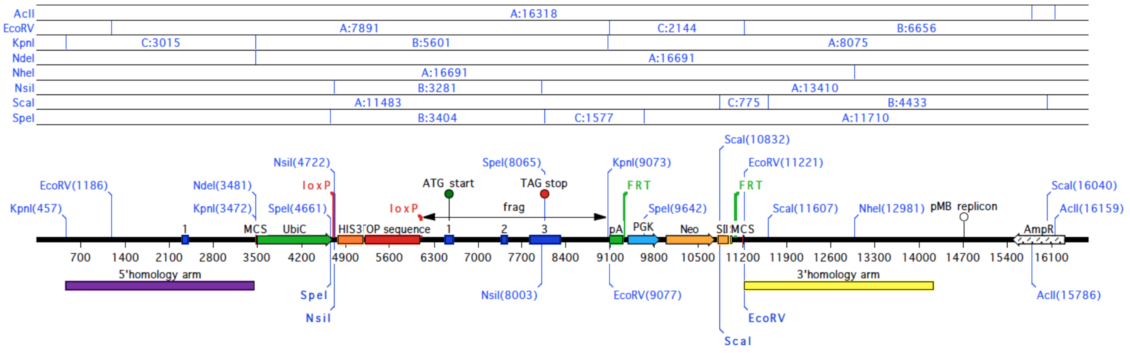

4.3 Restriction enzyme digest for electroporation

Finally, *targeting vector* was digested with AclI to obtain a suitable vector for electroporation into stem cells

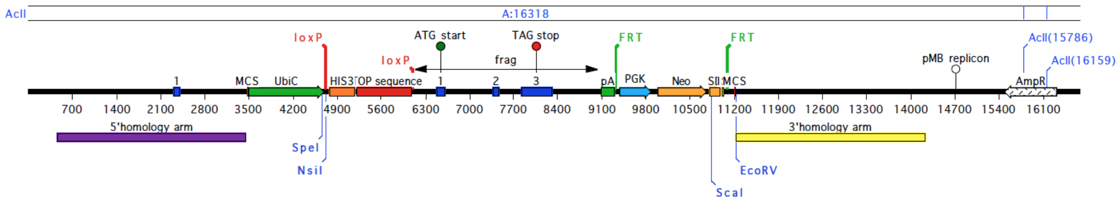

Supplementary material S4, Weight of selected tissues and organs, corresponding to the manuscript:

Ubiquitous transgenic overexpression of C-C chemokine ligand 2: a model to assess the combined effect of high energy intake and continuous low-grade inflammation?

Esther Rodríguez-Gallego, Marta Riera-Borrull, Anna Hernández-Aguilera, Roger Mariné-Casadó, Anna Rull, Raúl Beltrán-Debón, Fedra Luciano-Mateo, Javier A. Menéndez[1], Alejandro Vazquez-Martin[1], Juan J. Sirvent[2], Vicente Martín-Paredero[3], Angel L. Corbí[4], Elena Sierra-Filardi[4], Gerard Aragonès, Anabel García-Heredia, Jordi Camps, Carlos Alonso-Villaverde[5], Jorge Joven*

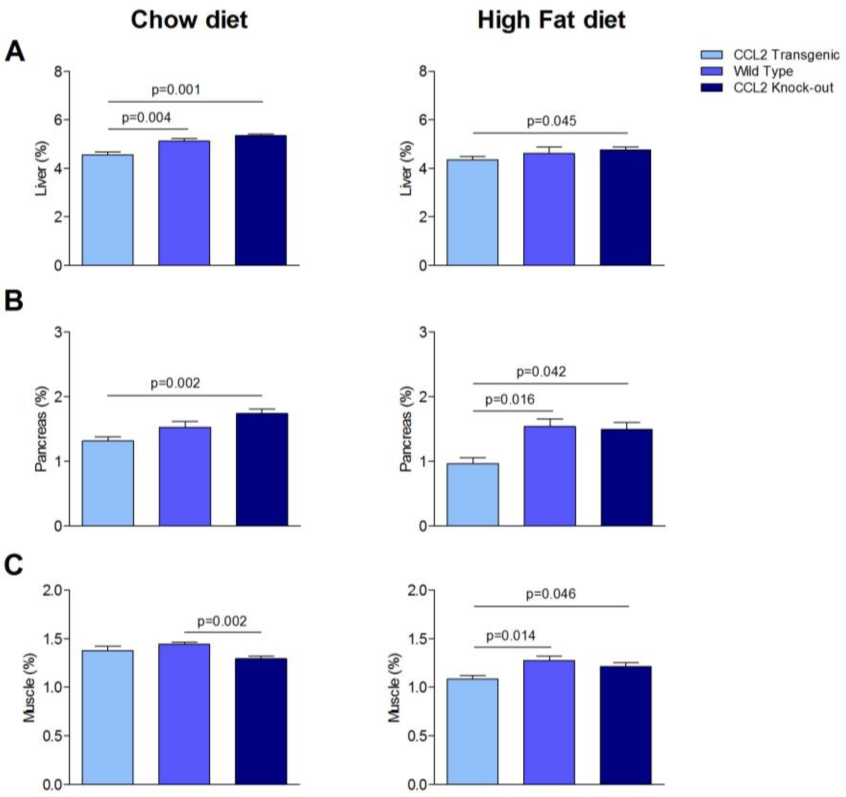
Chow diet
High Fat diet
A
B
C
CCL2 Transgenic
Wild Type
CCL2 Knock-out
Liver (%)
p=0.001
p=0.004
p=0.045
Pancreas (%)
p=0.002
p=0.042
p=0.016
Muscle (%)
p=0.002
p=0.046
p=0.014

Supplementary material S5, Weight of adipose tissues in the three strains after 14 weeks of a chow diet or high fat diet treatment.

Ubiquitous transgenic overexpression of C-C chemokine ligand 2: a model to assess the combined effect of high energy intake and continuous low-grade inflammation?

Esther Rodríguez-Gallego, Marta Riera-Borrull, Anna Hernández-Aguilera, Roger Mariné-Casadó, Anna Rull, Raúl Beltrán-Debón, Fedra Luciano-Mateo, Javier A. Menéndez[1], Alejandro Vazquez-Martin[1], Juan J. Sirvent[2], Vicente Martín-Paredero[3], Angel L. Corbí[4], Elena Sierra-Filardi[4], Gerard Aragonès, Anabel García-Heredia, Jordi Camps, Carlos Alonso-Villaverde[5], Jorge Joven*

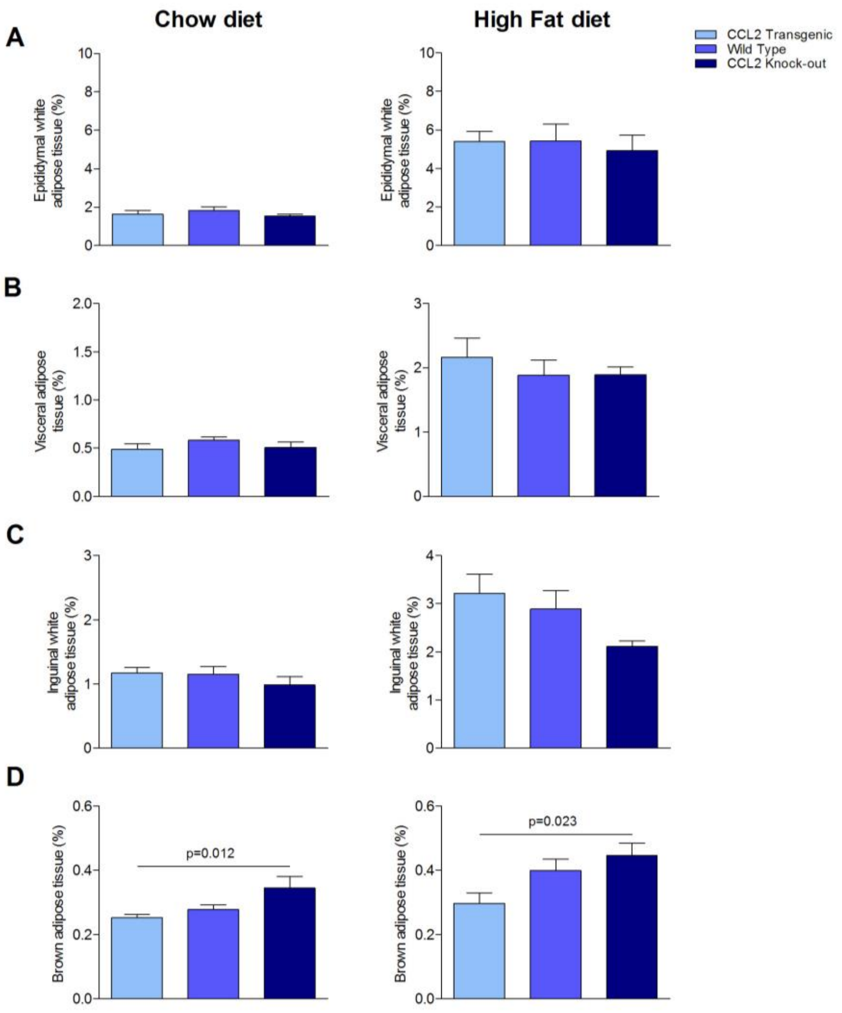

Supplementary material S6, disturbances in glucose metabolism, corresponding to the manuscript:

Ubiquitous transgenic overexpression of C-C chemokine ligand 2: a model to assess the combined effect of high energy intake and continuous low-grade inflammation?

Esther Rodríguez-Gallego, Marta Riera-Borrull, Anna Hernández-Aguilera, Roger Mariné-Casadó, Anna Rull, Raúl Beltrán-Debón, Fedra Luciano-Mateo, Javier A. Menéndez[1], Alejandro Vazquez-Martin[1], Juan J. Sirvent[2], Vicente Martín-Paredero[3], Angel L. Corbí[4], Elena Sierra-Filardi[4], Gerard Aragonès, Anabel García-Heredia, Jordi Camps, Carlos Alonso-Villaverde[5], Jorge Joven*

Oral glucose tolerance tests displayed a significant effect of high fat, high-cholesterol diet on insulin resistance even with a short-term manipulation. However, differences among strains fed the same diet were negligible either in 16 an 24 weeks old (A, B). Plasma glucose was higher in CCL2 overexpressors than in other strains and the effect of diet was also more evident (C). These differences disappeared when the dietary tratement was for 14 weeks (D). * P<0.05 with respect to relevant pair.

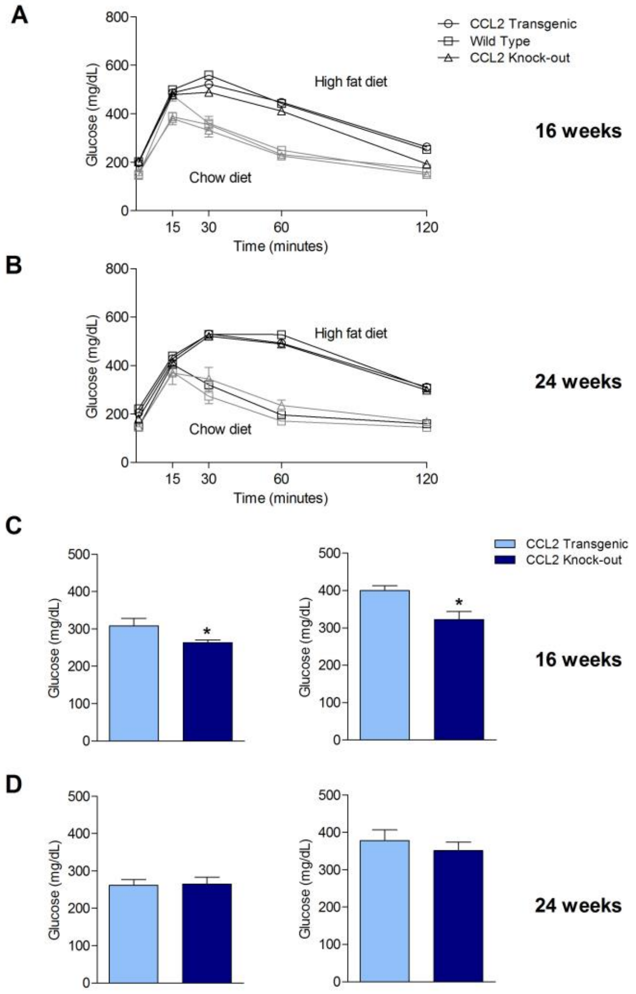

Supplementary material S7, expression of fatty acid synthase and AMPK in the liver, corresponding to the manuscript:

Ubiquitous transgenic overexpression of C-C chemokine ligand 2: a model to assess the combined effect of high energy intake and continuous low-grade inflammation?

Esther Rodríguez-Gallego, Marta Riera-Borrull, Anna Hernández-Aguilera, Roger Mariné-Casadó, Anna Rull, Raúl Beltrán-Debón, Fedra Luciano-Mateo, Javier A. Menéndez[1], Alejandro Vazquez-Martin[1], Juan J. Sirvent[2], Vicente Martín-Paredero[3], Angel L. Corbí[4], Elena Sierra-Filardi[4], Gerard Aragonès, Anabel García-Heredia, Jordi Camps, Carlos Alonso-Villaverde[5], Jorge Joven*

Representative immunoblottings in the liver of transgenic and KO mice fed chow diet (A,C,D) and high-fat diet (B,E, F) of FASN and activated AMPK and the respective calculations (C-F) .

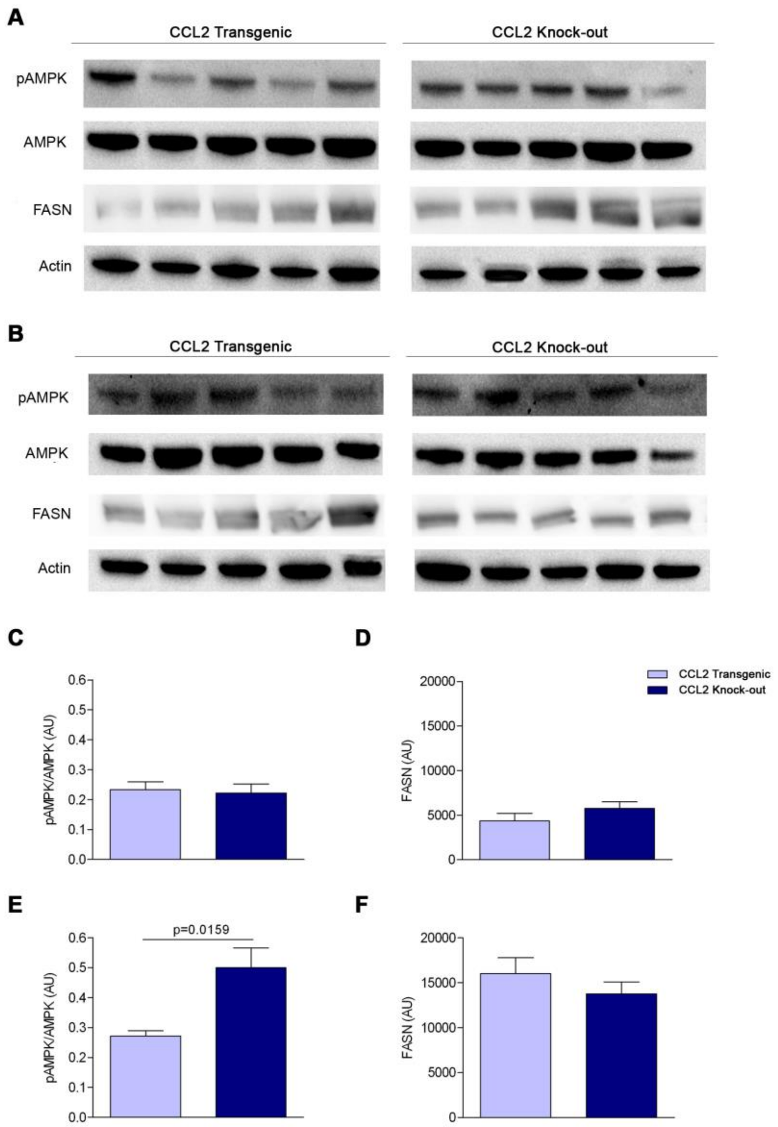
A
CCL2 Transgenic
CCL2 Knock-out
pAMPK
AMPK
FASN
Actin
B
CCL2 Transgenic
CCL2 Knock-out
pAMPK
AMPK
FASN
Actin
C
pAMPK/AMPK (AU)
D
FASN (AU)
CCL2 Transgenic
CCL2 Knock-out
E
p=0.0159
pAMPK/AMPK (AU)
F
FASN (AU)

Supplementary material S8 dietary fat and overexpression of CCL2 modify the size, number and morphology of liver macrophages also after 14 weeks of dietary treatment corresponding to the manuscript:

Ubiquitous transgenic overexpression of C-C chemokine ligand 2: a model to assess the combined effect of high energy intake and continuous low-grade inflammation?

Esther Rodríguez-Gallego, Marta Riera-Borrull, Anna Hernández-Aguilera, Roger Mariné-Casadó, Anna Rull, Raúl Beltrán-Debón, Fedra Luciano-Mateo, Javier A. Menéndez[1], Alejandro Vazquez-Martin[1], Juan J. Sirvent[2], Vicente Martín-Paredero[3], Angel L. Corbí[4], Elena Sierra-Filardi[4], Gerard Aragonès, Anabel García-Heredia, Jordi Camps, Carlos Alonso-Villaverde[5], Jorge Joven*

Dietary fat (right column) and CCL2 expression modify the size, number and morphology of liver macrophages with respect to those fed a chow diet (left column) as assessed with F4/80 staining. Values for stained area and length of macrophages (A-D) are illustrated with representative microphotographs from transgenic (E,F), WT (G,H) and KO mice (I,J).

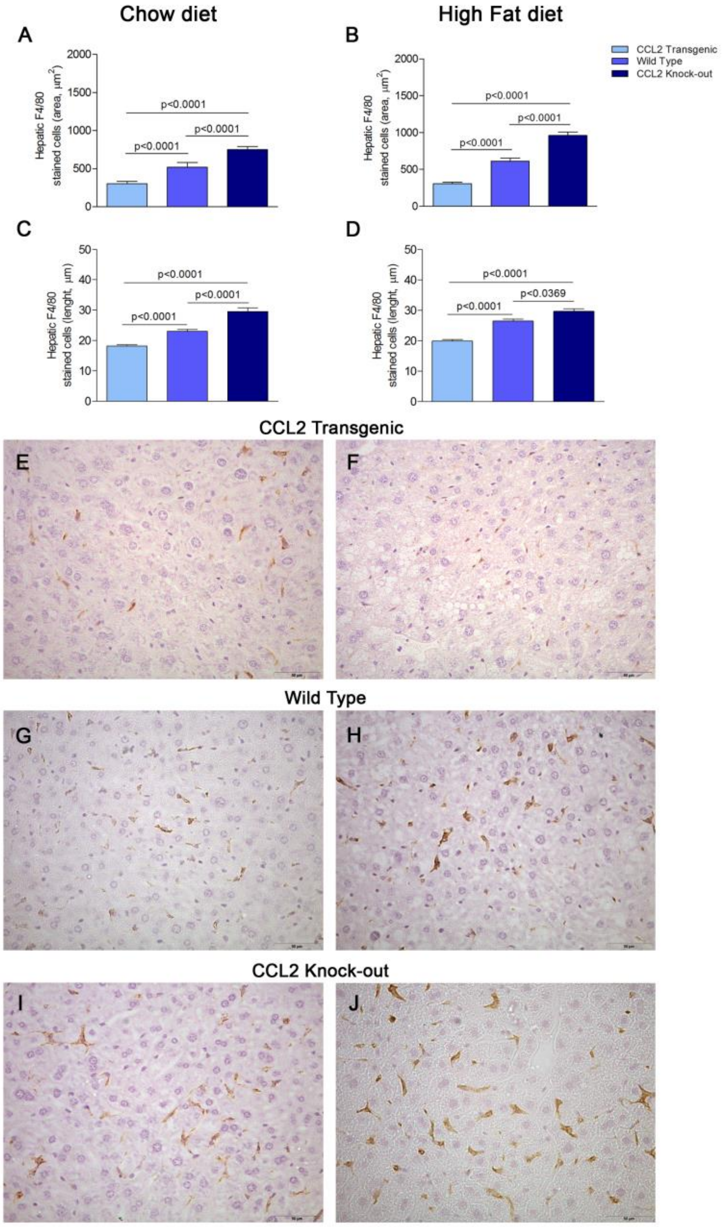

Chow diet
High Fat diet
A
B
CCL2 Transgenic
Wild Type
CCL2 Knock-out
Hepatic F4/80 stained cells (area, μm2)
p<0.0001
C
D
Hepatic F4/80 stained cells (lenght, μm)
p<0.0369
CCL2 Transgenic
E
F
Wild Type
G
H
CCL2 Knock-out
I
J

Supplementary material S9, Transgenic mice that overexpress CCL2 die prematurely when fed high-fat diet, corresponding to the manuscript:

Ubiquitous transgenic overexpression of C-C chemokine ligand 2: a model to assess the combined effect of high energy intake and continuous low-grade inflammation?

Esther Rodríguez-Gallego, Marta Riera-Borrull, Anna Hernández-Aguilera, Roger Mariné-Casadó, Anna Rull, Raúl Beltrán-Debón, Fedra Luciano-Mateo, Javier A. Menéndez[1], Alejandro Vazquez-Martin[1], Juan J. Sirvent[2], Vicente Martín-Paredero[3], Angel L. Corbí[4], Elena Sierra-Filardi[4], Gerard Aragonès, Anabel García-Heredia, Jordi Camps, Carlos Alonso-Villaverde[5], Jorge Joven*

Transgenic mice fed high-fat diet died prematurely after a progressive decrease in weight and activity (A,B). The size of the spleen in transgenic animals was higher than in controls and there were abundant megakariocytes (C, D). The livers were also higher in transgenic mice where steatosis and signs of regenerative tissue and apoptotic nuclei were numerous (E,F)

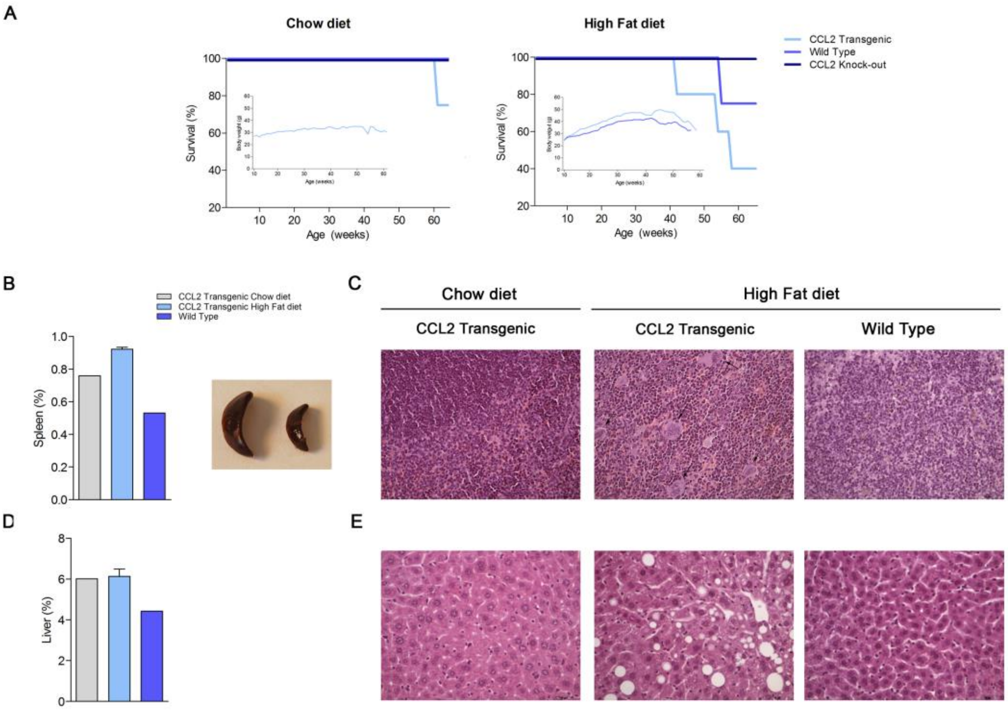
A
Chow diet
High Fat diet
CCL2 Transgenic
Wild Type
CCL2 Knock-out
Survival (%)
Age (weeks)
B
CCL2 Transgenic Chow diet
CCL2 Transgenic High Fat diet
Wild Type
Spleen (%)
C
Chow diet
High Fat diet
CCL2 Transgenic
CCL2 Transgenic
Wild Type
D
Liver (%)
E
